# Association of Maternal Use of Benzodiazepines and Z-Hypnotics During Pregnancy With Motor and Communication Skills and Attention-Deficit/Hyperactivity Disorder Symptoms in Preschoolers

**DOI:** 10.1001/jamanetworkopen.2019.1435

**Published:** 2019-04-05

**Authors:** Angela Lupattelli, Cristina D. Chambers, Gretchen Bandoli, Marte Handal, Svetlana Skurtveit, Hedvig Nordeng

**Affiliations:** 1 PharmacoEpidemiology and Drug Safety Research Group, Department of Pharmacy, University of Oslo, Oslo, Norway; 2 PharmaTox Strategic Research Initiative, Faculty of Mathematics and Natural Sciences, University of Oslo, Oslo, Norway; 3 Department of Family Medicine and Public Health, University of California, San Diego, La Jolla; 4 Department of Pediatrics, University of California, San Diego, La Jolla; 5 Department of Mental Disorders, Norwegian Institute of Public Health, Oslo, Norway; 6 Department of Child Health and Development, Norwegian Institute of Public Health, Oslo, Norway

## Abstract

**Importance:**

The reproductive safety of benzodiazepine/z-hypnotic exposure on child longer-term developmental risks remains unresolved.

**Objective:**

To quantify the association of motor, communication, and attention-deficit/hyperactivity disorder (ADHD) symptoms in preschoolers with gestational benzodiazepine/z-hypnotic exposure by timing and duration and coexposure to opioids or antidepressants.

**Design, Setting, and Participants:**

Nationwide, population-based Norwegian Mother and Child Cohort Study, recruiting pregnant women from 1999 to 2008, with child follow-up from ages 6, 18, and 36 months to ages 5, 7, and 8 years. Follow-up of teenagers is ongoing. The study included women with depressive/anxiety (n = 4195), sleeping (n = 5260), or pain-related (n = 26 631) disorders before and/or during pregnancy.

**Exposures:**

For the timing analyses, children exposed to benzodiazepines/z-hypnotics in midpregnancy (weeks 17-28) or late pregnancy (week 29 or later) vs those born to nonmedicated women. For the duration and coexposure analyses, benzodiazepine/z-hypnotic treatment for multiple 4-week intervals vs 1 and co-use of benzodiazepine/z-hypnotic with opioids or antidepressants vs sole benzodiazepine/z-hypnotic use.

**Main Outcomes and Measures:**

Parent-reported motor and communication skills (Ages and Stages Questionnaires) and ADHD symptoms (Conners’ Parent Rating Scale–Revised) at child median age of 5.1 years (interquartile range, 5.0-5.3 years) as standardized mean scores. General linear propensity score–adjusted and marginal structural models were fitted. Analyses were stratified by maternal disorder.

**Results:**

Of 41 146 eligible pregnancy-child dyads, 36 086 children (18 330 boys and 17 756 girls) were included, of whom 283 (0.8%) were prenatally exposed to benzodiazepines/z-hypnotics (134 in the depressive/anxiety, 60 in the sleeping, and 89 in the pain-related disorders). There was no increased risk for greater ADHD symptoms or fine motor deficits after intrauterine benzodiazepine/z-hypnotic exposure at different time points. Children born to women with depressive/anxiety disorders who took benzodiazepines/z-hypnotics in late pregnancy had greater gross motor (weighted β, 0.67; 95% CI, 0.21-1.13) and communication (weighted β, 0.35; 95% CI, 0.04-0.65) deficits than unexposed children. There was no evidence for substantial duration or coexposure associations.

**Conclusions and Relevance:**

These findings suggest no substantial detrimental risk on child fine motor and ADHD symptoms after prenatal benzodiazepine/z-hypnotic exposure alone or in combination with opioids or antidepressants. Residual confounding by indication and/or a higher drug dose regimen among women with anxiety/depression may explain the moderate association of gross motor and communication deficits with late-pregnancy benzodiazepine/z-hypnotic use.

## Key Points

**Question:**

Is the association of prenatal benzodiazepine/z-hypnotic exposure with child developmental risks different according to timing of exposure, duration, or coexposure to opioids or antidepressants?

**Findings:**

Among 41 146 pregnancy-child dyads in this cohort study, a moderate association between benzodiazepine/z-hypnotic exposure in late pregnancy and greater gross motor and communication deficits in children born to women with depressive/anxiety disorders were observed, but not to the extent that the impairment was of clinical relevance. There was no evidence for duration or coexposure associations on all outcomes.

**Meaning:**

These findings show no clinically relevant detrimental risk of prenatal benzodiazepine/z-hypnotic exposure on motor, communication, and attention-deficit/hyperactivity disorder outcomes in preschoolers.

## Teaser

This cohort study evaluates the association of motor, communication, and attention-deficit/hyperactivity disorder (ADHD) symptoms in preschoolers with gestational benzodiazepine/z-hypnotic exposure by timing and duration and coexposure to opioids or antidepressants.

## Introduction

Up to 15% of pregnant women have an anxiety disorder, often comorbid with depression,^1,2^ and benzodiazepines are at times required given their anxiolytic and sedative effects.^3^ The z-hypnotics are benzodiazepine-like drugs that can be used for treatment of insomnia, a common symptom of generalized anxiety.^4^ During pregnancy, use of benzodiazepines and/or z-hypnotics is in the range of 1% to 4%,^5,6,7^ and both medications may interfere with fetal brain maturation because of their shared modulating activity on the γ-aminobutyric acid receptor.^8,9^ Nevertheless, their safety in relation to offspring longer-term outcomes has so far received limited attention.

Associations between prenatal benzodiazepine exposure and gross motor and fine motor impairment have been observed in toddlers, although the gross motor delay resolved as children grew older.^10,11^ Confounding by indication, along with small sample size and short follow-up, constitutes a major drawback of this prior research.^10,11,12^ Three more recent, methodologically sound studies^13,14,15^ found no greater risk for lower language competence or externalizing or aggressive behaviors in offspring at ages 3 and 6 years, although a small risk (β, 0.26; 95% CI, 0.00-0.52) of internalizing behaviors was noted after in utero benzodiazepine exposure.^13^

Both benzodiazepines and z-hypnotics are intermittently used during gestation,^5^ but it remains unresolved whether early or late exposure or rather duration of pharmacotherapy confers different longer-term risks in offspring. Because use of benzodiazepines and z-hypnotics often occurs with greater concurrent use of opioid analgesics or antidepressants in pregnancy,^16^ a better understanding of the association between this coexposure and child risk is also crucial.

Herein, we sought to quantify the association of time-varying benzodiazepine/z-hypnotic exposure during pregnancy with child gross motor and fine motor skills, communication, and attention-deficit/hyperactivity disorder (ADHD) traits by age 5 years. In additional subanalyses, we aimed to estimate the association of duration of benzodiazepine/z-hypnotic exposure and co-use of opioid analgesics or antidepressants with these outcomes. We hypothesized that there would be no detrimental risk of benzodiazepine/z-hypnotic exposure in pregnancy on child motor, communication, and ADHD outcomes.

## Methods

Data from the nationwide, population-based Norwegian Mother and Child Cohort Study (MoBa) were linked to the Medical Birth Registry of Norway (MBRN) via the women’s personal identification numbers. The MoBa is a prospective population-based pregnancy cohort study conducted by the Norwegian Institute of Public Health.^17,18^ Participants were recruited from all over Norway from 1999 to 2008 through a postal invitation in connection with publicly offered routine ultrasonography at 17 to 18 weeks’ gestation. Data were gathered prospectively via 2 prenatal self-administered questionnaires at week 17 (questionnaire 1) and week 30 (questionnaire 3). Follow-up questionnaires on maternal and child health were sent to mothers when the child was age 6 months (questionnaire 4), 18 months (questionnaire 5), and 36 months (questionnaire 6) to age 5 years (questionnaire 7), 7 years, and 8 years and up to teenage years.^19^ Follow-up of children started in 1999 and is still ongoing in teenagers. Prospective fathers also completed 1 prenatal questionnaire. The present study is based on version 9 of the quality-assured data files, which include complete follow-up data at child age 5 years. The cohort now includes 114 500 children, 95 200 mothers, and 75 200 fathers.^17^ The participation rate for all invited pregnancies is 41%. Of those agreeing to participate, the response rate ranges from 95% (questionnaire 1) and 92% (questionnaire 3) to 77% (questionnaire 5).^18^ This study followed the Strengthening the Reporting of Observational Studies in Epidemiology (STROBE) reporting guideline.

The MoBa obtained a license from the Norwegian Data Inspectorate and approval from the Regional Committee for Medical Research Ethics. All individuals provided written informed consent before participation. The MBRN is based on compulsory notification of all live births, stillbirths, and induced abortions.^20^ The Figure shows the exclusion criteria to achieve the final study population.

**Figure.**
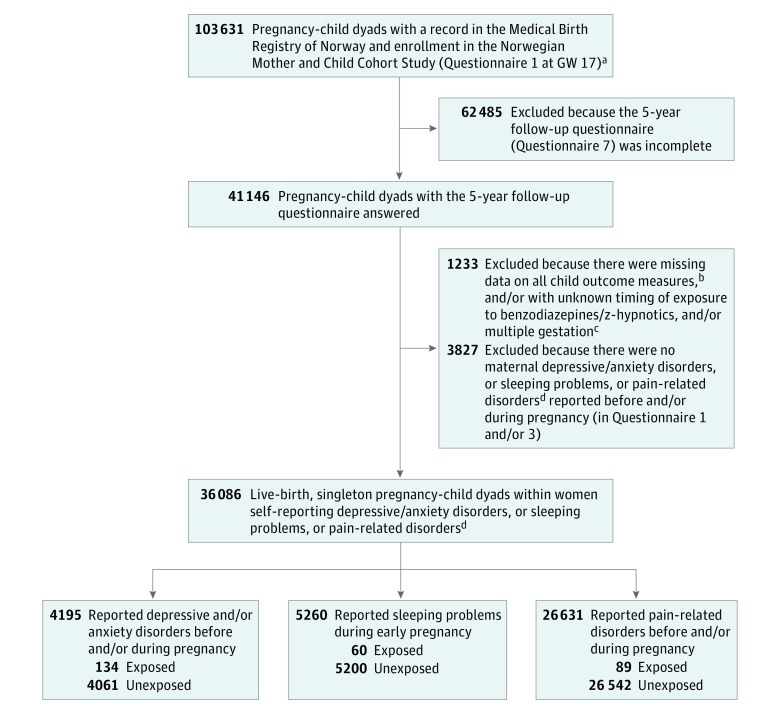
Flowchart to Achieve the Final Study Population Conditions of exclusion may overlap. ^a^Questionnaire 1 is the first Norwegian Mother and Child Cohort Study questionnaire, completed at 17 gestational weeks (GW). Completion of questionnaire 1 implied enrollment in the study. ^b^Missing information on all Ages and Stages Questionnaires subscales and on the Conners’ Parent Rating Scale–Revised. ^c^Indicates 1299 twin and 14 triplet pregnancies. ^d^Includes long-term (ie, arthritis, sciatica, fibromyalgia, headache, and migraine) pain-related conditions before and/or during pregnancy and acute pain-related conditions (ie, pelvic girdle, back, groin, and muscle/joint pains) during pregnancy.

### Maternal Disorders

We included pregnancies in women having an underlying indication for treatment with benzodiazepines/z-hypnotics (ie, depressive and/or anxiety disorders).^3,21^ Because z-hypnotics are used to treat sleeping problems and benzodiazepine may be coprescribed for pain management,^22^ these 2 indications were additionally considered. In questionnaires 1 and 3 of the MoBa,^19^ women were presented a list of previous and/or concurrent illnesses and could indicate whether they have had (1) depression or anxiety or other mental disorders (hereafter “depressive/anxiety disorders” because these were the most commonly reported) before and/or during pregnancy, (2) sleeping problems during early pregnancy, or (3) long-term or acute pain-related conditions before/during or only during pregnancy, respectively (Figure). In case of comorbidity, women were assigned a primary underlying disorder based on the above hierarchy. We conducted all analyses separately in each maternal disorder stratum. Maternal depressive and anxiety symptom severity was measured via the short versions of the Hopkins Symptom Checklist 25 (SCL-25) at weeks 17 (5 items [SCL-5]) and 30 (8 items [SCL-8]).^23,24^ More information is provided in the eAppendix in the Supplement.

### Exposures

Questionnaires 1, 3, and 4 provided information about benzodiazepine and z-hypnotic exposure.^19^ Women reported the name of the medication taken along with the timing of use (6 months before pregnancy and during pregnancy by 4-week intervals). On the basis of the Anatomical Therapeutic Chemical (ATC) classification system,^25^ benzodiazepines included drugs within the ATC groups N05BA (diazepam, oxazepam, and alprazolam), N05CD (nitrazepam, midazolam hydrochloride, and flunitrazepam), and N03AE01 (clonazepam). The z-hypnotics included zopiclone and zolpidem (N05CF). Due to similar mechanisms of actions, benzodiazepines and z-hypnotics were studied as 1 group and separate classes.

To explore the temporal sequence between measurement of depressive/anxiety symptoms and drug use, we defined the primary exposure windows as early pregnancy (weeks 0-16), midpregnancy (weeks 17-28), and late pregnancy (week 29 to delivery) (eFigure 1 in the Supplement). Duration of benzodiazepine and z-hypnotic exposure was defined according to whether a single or multiple 4-week intervals were checked in the questionnaires. Women were classified as exposed if they reported use of benzodiazepine and/or z-hypnotic during these periods. We defined coexposure to an opioid (ATC N02A) or an antidepressant (ATC N06A) as reported co-use of each of these medication classes with benzodiazepine/z-hypnotic during gestation. In the coexposure analysis, the reference group consisted of pregnancies exposed to benzodiazepines/z-hypnotics but not to opioids or antidepressants during pregnancy. The timing analyses were conducted separately in each maternal disorder stratum; the duration and coexposure analyses were solely performed in women with depressive/anxiety disorders.

### Outcomes

Child outcomes were parent reported via completion of widely used, validated diagnostic measures of child development and behavior, including the Ages and Stages Questionnaires (ASQ) and the Conners’ Parent Rating Scale–Revised (CPRS-R).^26,27,28^ The MoBa included selected ASQ items representing the gross motor, fine motor, and communication developmental domains (6 items per domain). Mothers were asked to rate whether each item reflected their child’s motor skills and ability to understand and communicate. Child ADHD traits of inattention and hyperactivity/impulsivity were measured by 12 CPRS-R items. Mothers were asked to rate whether each item reflected their child’s behavior in the last 6 months. The ASQ and CPRS-R items and related scoring are shown in eFigure 2 in the Supplement. For each domain within the scales, the mean scores were calculated and standardized. Higher *z* scores indicated greater endorsement of each domain (eg, greater fine motor deficit). In this study, the internal consistency was 0.6 to 0.7 for the ASQ domains and 0.9 for the CPRS-R.

### Covariates

We identified a sufficient set of confounders with the aid of directed acyclic graphs and subject knowledge.^29^ These included the following: maternal folate intake, parity, and marital status as ascertained from the MBRN; body mass index, gross yearly income, smoking and alcohol use in pregnancy, and maternal and paternal education as reported in the MoBa questionnaires; self-reported comedication with opioid analgesics, acetaminophen, nonsteroidal anti-inflammatory drugs, other psychotropics (ie, antipsychotics, antiepileptics, and antidepressants), and sedating antihistamines; severity of maternal depressive and anxiety symptoms in pregnancy as measured by the SCL-5 or SCL-8 in the MoBa; lifetime history of major depression measured via 5 key depressive symptoms closely corresponding to the *Diagnostic and Statistical Manual of Mental Disorders* (Third Edition) criteria for lifetime major depression^30^; presence and painfulness of maternal adverse life events close to the pregnancy period as measured in questionnaire 3; and an obstetric comorbidity index based on MBRN records.^31^

Postnatal and other parental factors were taken into account under alternate model specifications in the timing analyses (eTable 1 in the Supplement). Further information on covariates is provided in the eAppendix in the Supplement.

### Statistical Analysis

#### Timing Analyses

To estimate associations by timing of exposure, we fit marginal structural models with 2 time points to account for (1) time-varying benzodiazepine/z-hypnotic exposure, (2) time-varying confounders (ie, depressive and anxiety symptoms in pregnancy and comedication with opioids, antidepressants, sedatives, antihistamines, or acetaminophen), and (3) loss to follow-up.^32,33^ We estimated the probability of benzodiazepine/z-hypnotic treatment using a pooled logistic regression in which the outcome was current treatment with a benzodiazepine/z-hypnotic in midpregnancy or late pregnancy and covariates were maternal baseline factors, time-varying and time-fixed confounders, and benzodiazepine/z-hypnotic treatment in gestational weeks 0 to 16 (model 1 in eTable 1 in the Supplement). We also calculated the probability of remaining in the study given maternal baseline covariates and then derived stabilized inverse probability of treatment weighting (IPTW) and inverse probability of censoring weighting (IPCW) for each pregnancy at each time point. Generalized linear models with robust standard errors were fit applying the IPTW and the composite IPTW*IPCW. To further examine confounding by indication, we conducted separate analyses for each maternal disorder stratum. Analyses by medication class were also performed.

#### Duration and Coexposure Analyses

Because women in the depressive/anxiety disorder stratum were most often coexposed to an opioid or antidepressant and treated for longer periods, we determined duration and coexposure associations solely in this stratum by fitting crude and propensity score–adjusted generalized linear models with robust standard errors. Logistic regression models were first fit to estimate the probability of (1) exposure to benzodiazepine/z-hypnotic in 2 or more intervals during pregnancy relative to 1 interval and (2) co-use of benzodiazepine/z-hypnotic-antidepressant or benzodiazepine/z-hypnotic-opioid during pregnancy relative to benzodiazepine/z-hypnotic alone given a modified set of sufficient confounders (eAppendix in the Supplement).

The crude and adjusted β coefficients with 95% CIs represent the standardized mean difference in the developmental outcomes between children according to the various exposure definitions. Power analysis for the various exposure windows is summarized in eTable 2 and eTable 3 in the Supplement. The study had enough statistical power to detect clinically relevant effect sizes (Cohen *d* > 1.00) or smaller in most analytical scenarios.

#### Missing Data and Multiple Imputation

Up to 16.5% of the pregnancies had missing values in at least 1 of the sufficient confounders. Under the assumption that data were missing at random, we imputed incomplete data via multiple imputation (eAppendix in the Supplement).^34,35,36^

#### Sensitivity Analyses

We conducted a number of sensitivity analyses to assess the robustness of our findings as described in the eAppendix in the Supplement. To verify the validity of the outcome measures, we evaluated the strength of the following associations: (1) child diagnosis of language or motor delay/clumsy at age 5 years with communication or motor skills on the ASQ and (2) maternal and paternal ADHD traits with child’s ADHD traits on the CPRS-R. As a negative control, we used children born to women who took benzodiazepines/z-hypnotics in the 6-month period before pregnancy but not during pregnancy. We conducted probabilistic bias analyses to correct for exposure misclassification, unmeasured confounding, and random error by specifying trapezoidal distributions of the bias parameters (eAppendix in the Supplement).^37,38^ To address the role of chance, we reestimated the association measures of the main analyses with the corresponding 99% CIs. All statistical analyses were performed using a software program (Stata, version 15; StataCorp LP).

## Results

Of 41 146 eligible pregnancy-child dyads, the study population comprised 36 086 children (18 330 boys and 17 756 girls) of 32 799 mothers (Figure). Relative to women who remained in the study, those lost to follow-up between childbirth and 5 years’ postpartum more often had unfavorable correlates (eg, lower education and income, more severe antenatal depressive symptoms, and smoking in pregnancy). Use of benzodiazepines/z-hypnotics in gestation was not associated with loss to follow-up.

Depressive/anxiety disorders and sleeping problems constituted the primary maternal disorder for 11.6% (n = 4195) and 14.6% (n = 5260) of the pregnancies, respectively, and included pain-related disorders for the remainder (73.8% [n = 26 631]) (Figure). Of the women with depressive/anxiety disorders, most reported depression before/during pregnancy either alone (n = 2437) or comorbid with anxiety and/or other mental illnesses (n = 1057). Anxiety or other mental illness alone was reported by 435 and 220 women, respectively. Baseline characteristics of the sample by benzodiazepine/z-hypnotic exposure are listed during pregnancy overall in Table 1 and by maternal primary disorder in eTable 4 in the Supplement. The distributions of missing data on confounders by exposure status in pregnancy are shown in eFigures 3, 4, and 5 in the Supplement. The median gestational weeks when the 2 prenatal questionnaires were completed were 16.9 (interquartile range [IQR], 15.4-18.7) and 30.1 (IQR, 29.0-31.4).

**Table 1.** Cohort Characteristics by Exposure to Benzodiazepines/Z-Hypnotics During Pregnancy Among 36 086 Children

Variable	Benzodiazepine/Z-Hypnotic Exposure During Pregnancy^a^	
	No (n = 35 803)	Yes (n = 283)
**Maternal Sociodemographics and Lifestyle**		
Age, mean (SD), y	30.6 (4.4)	31.7 (4.4)
BMI at conception, mean (SD)	24.0 (4.2)	23.8 (4.2)
Primiparous, No. (%)	16 952 (47.3)	143 (50.5)
Married/cohabiting, No. (%)	34 571 (96.6)	258 (91.2)
Educational level, No. (%)^b^		
University/college	25 646 (71.6)	211 (74.6)
Less than university/college	10 004 (27.9)	71 (25.1)
Gross yearly income, No. (%)^c^		
Average	26 347 (73.6)	204 (72.1)
Low	4011 (11.2)	33 (11.7)
High	4515 (12.6)	38 (13.4)
Smoking status (yes) at wk 30, No. (%)	1634 (4.6)	34 (12.0)
Alcohol use in pregnancy, No. (%)		
No/very limited use	31 628 (88.3)	222 (78.4)
Medium use	3354 (9.4)	46 (16.3)
Weekly use	298 (0.8)	12 (4.2)
Folate intake (yes), No. (%)^d^	31 499 (88.0)	250 (88.3)
**Maternal Health**		
Comorbidity index, mean (SD)* z* score	0.02 (1.01)	0.35 (1.24)
Lifetime history of major depression (yes), No. (%)^e^	2236 (6.2)	54 (19.1)
Depressive/anxiety symptoms during pregnancy, mean (SD) *z* score		
SCL-5 at wk 17	−0.01 (0.99)	0.91 (1.81)
SCL-8 at wk 30	−0.01 (0.99)	0.93 (1.71)
Emotional stability trait (range, 1-5), mean (SD)^f^	2.7 (0.5)	2.9 (0.5)
Lifetime adverse events at baseline, No. (%)^g^		
None or ≥1 event but not painful	21 967 (61.4)	110 (38.9)
≥1 Event, painful	8165 (22.8)	94 (33.2)
≥1 Event, very painful	4141 (11.6)	66 (23.3)
Comedication in pregnancy (yes), No. (%)		
Antidepressants	361 (1.0)	55 (19.4)
Antipsychotics	284 (0.8)	17 (6.0)
Opioid analgesics	707 (2.0)	35 (12.4)
Antiepileptic drugs	133 (0.4)	6 (2.1)
Nonsteroidal anti-inflammatory drugs	2369 (6.6)	41 (14.5)
Acetaminophen	17 571 (49.1)	197 (69.6)
Sedating antihistamines	193 (0.5)	30 (10.6)
Illicit substance use (yes), No. (%)^h^	202 (0.6)	14 (4.9)
**Child and Postpartum Characteristics**		
Breastfeeding months up to child age 6 mo, mean (SD)	5.7 (2.5)	5.5 (2.5)
Infant sex (male), No. (%)	18 185 (50.8)	145 (51.2)
Any malformation (yes), No. (%)	1745 (4.9)	13 (4.6)
Premature birth (yes), No. (%)	1617 (4.5)	17 (6.0)
Nursery/daycare attendance between ages 1-5 y, No. (%)		
Never	5229 (14.6)	50 (17.7)
Any time	25 710 (71.8)	198 (70.0)
Always	4864 (13.6)	35 (12.4)
No. of postnatal maternal adverse events, mean (SD)^i^		
Between 0-3 y postpartum	0.59 (0.95)	0.86 (1.12)
Between 4-5 y postpartum	0.81 (1.06)	1.28 (1.38)
Postnatal depressive/anxiety symptoms, mean (SD) *z* score		
SCL-8 average between 0.5-5 y postpartum	−0.01 (0.99)	0.81 (1.45)
SCL-8 specifically at 5 y postpartum	−0.01 (1.00)	0.53 (1.33)
Maternal ADHD symptoms at 3 y postpartum, No. (%)		
None	26 399 (73.7)	190 (67.1)
Mild	2272 (6.3)	32 (11.3)
Moderate to severe	350 (1.0)	8 (2.8)
Parents’ positive involvement with children, mean (SD) *z* score	0.00 (1.00)	−0.06 (1.09)
**Paternal Characteristics, No. (%)**		
Age, y		
<25	1255 (3.5)	5 (1.8)
25-39	30 786 (86.0)	227 (80.2)
40-49	3422 (9.6)	43 (15.2)
>49	257 (0.7)	3 (1.1)
Educational level		
University/college	19 189 (53.6)	147 (51.9)
Less than university/college	16 370 (45.7)	132 (46.6)
Sleeping problems (yes)	1739 (4.9)	23 (8.1)
Mental illness (yes)	390 (1.1)	4 (1.4)
Paternal ADHD symptoms at time of pregnancy		
None	13 660 (38.2)	101 (35.7)
Mild	2201 (6.1)	22 (7.8)
Moderate to severe	258 (0.7)	3 (1.1)

Abbreviations: ADHD, attention-deficit/hyperactive disorder; BMI, body mass index (calculated as weight in kilograms divided by height in meters squared); SCL, short versions of the Hopkins Symptom Checklist.

^a^ Numbers may not add up to totals due to missing values, ranging from 0.4% to 0.7% (maternal/paternal educational level), to 1.5% to 1.9% (BMI and alcohol use in pregnancy), and 2.1% to 2.7% (gross yearly income, smoking status, and lifetime history of major depression). For the prenatal SCL-5 and SCL-8, missing values were 2.8% and 4.6%, respectively; missing values for lifetime adverse events at baseline were 4.9%. Information on maternal and paternal ADHD and parenting was available for 30% to 65% of the study population because the instruments were only present in later versions of the Norwegian Mother and Child Cohort Study questionnaire. The paternal questionnaire was available for 30 326 mother-child dyads (84.0%).

^b^ Ongoing or completed educational level.

^c^ Average is $14 800 to $49 900, low is $14 800 or less, and high is at least $50 000.

^d^ Folate before and/or during first trimester.

^e^ Defined as Kendler Lifetime Major Depression Scale score of 3 or more simultaneous depressive symptoms of duration of more than 2 weeks.

^f^ As measured by the International Personality Item Pool Big-Five Factor Markers.

^g^ Adverse life events in the perinatal period (ie, from 7 months before pregnancy to week 30 of pregnancy).

^h^ Before and/or during pregnancy.

^i^ Number of adverse life events in the early and late postnatal period, with no severity specification.

Gestational exposure to any benzodiazepine/z-hypnotic occurred in 283 pregnancies (0.8%) (134 in the depressive/anxiety, 60 in the sleeping, and 89 in the pain-related disorders). Benzodiazepines-anxiolytics (n = 147 [mainly diazepam and oxazepam]) and z-hypnotics (n = 133 [mainly zopiclone]) were the most common exposures. The highest proportion of pharmacotherapy, coexposure to benzodiazepine/z-hypnotic-opioid or benzodiazepine/z-hypnotic-antidepressant, and longer treatment duration was in women with depressive/anxiety disorders (eTable 2 and eTable 3 in the Supplement).

### Associations by Timing of Exposure

Child developmental outcomes were assessed by a median age of 5.1 years (IQR, 5.0-5.3 years). Benzodiazepine/z-hypnotic exposure at different time points in pregnancy did not pose any increased risk for greater fine motor deficits or ADHD traits in offspring (Table 2). Children of mothers with depressive/anxiety disorders exposed to benzodiazepines/z-hypnotics in late pregnancy had greater gross motor deficits (weighted β, 0.67; 95% CI, 0.21-1.13) than unexposed children in the time window. This association was only present among boys (weighted β, 0.91; 95% CI, 0.47-1.35) (eAppendix in the Supplement) and was observed for benzodiazepine and z-hypnotic monotherapy exposure. A small size association was also present between benzodiazepine/z-hypnotic use in late pregnancy and greater communication deficits (weighted β, 0.35; 95% CI, 0.04-0.65), mainly driven by z-hypnotic exposure (Table 2 and Table 3). These associations were not evident in the sleeping and pain-related disorder strata; in contrast, an inverse association was observed in these strata between benzodiazepine/z-hypnotic exposure and child motor skills (Table 2). The characteristics of the estimated weights are listed in eTable 5 in the Supplement. Adjusting for loss to follow-up did not materially change the main results (eTable 6 in the Supplement).

**Table 2.** Associations of Timing of Benzodiazepine/Z-Hypnotic Exposure in Pregnancy With Child Outcomes by Maternal Underlying Disorder

Variable	No.^a^	Depressive/Anxiety Disorders (n = 4195)		No.^a^	Sleeping Problems (n = 5260)		No.^a^	Pain-Related Disorders (n = 26 631)	
		Crude β (95% CI)	Weighted β (95% CI)^b^^,^^c^		Crude β (95% CI)	Weighted β (95% CI)^b^^,^^c^		Crude β (95% CI)	Weighted β (95% CI)^b^^,^^c^
**ASQ, Gross Motor Skills**									
Exposed, midpregnancy	55	0.03 (−0.25 to 0.31)	−0.19 (−0.55 to 0.18)	19	−0.39 (−0.51 to −0.26)	−0.30 (−0.52 to −0.09)	23	0.21 (−0.28 to 0.71)	0.15 (−0.70 to 1.01)
Exposed, late pregnancy	50	0.28 (−0.06 to 0.62)	0.67 (0.21 to 1.13)	17	−0.28 (−0.60 to 0.05)	−0.19 (−0.56 to 0.18)	24	−0.14 (−0.44 to 0.16)	−0.06 (−0.67 to 0.56)
**ASQ, Fine Motor Skills**									
Exposed, midpregnancy	55	0.02 (−0.46 to 0.50)	0.04 (−0.44 to 0.51)	19	−0.07 (−0.48 to 0.33)	−0.22 (−0.69 to 0.25)	24	0.11 (−0.28 to 0.49)	0.06 (−0.32 to 0.45)
Exposed, late pregnancy	49	0.55 (−0.11 to 1.22)	0.52 (−0.11 to 1.16)	17	−0.11 (−0.51 to 0.29)	0.08 (−0.58 to 0.73)	25	−0.32 (−0.51 to −0.12)	−0.45 (−0.69 to −0.21)
**ASQ, Communication Skills**									
Exposed, midpregnancy	54	0.16 (−0.12 to 0.44)	−0.11 (−0.40 to 0.19)	17	0.06 (−0.30 to 0.43)	0.21 (−0.28 to 0.69)	25	−0.14 (−0.47 to 0.19)	−0.17 (−0.57 to 0.23)
Exposed, late pregnancy	47	0.26 (−0.05 to 0.58)	0.35 (0.04 to 0.65)	16	−0.10 (−0.45 to 0.25)	−0.26 (−0.62 to 0.10)	24	−0.14 (−0.41 to 0.14)	−0.06 (−0.46 to 0.33)
**CPRS-R, ADHD Traits**									
Exposed, midpregnancy	54	0.20 (−0.13 to 0.52)	−0.04 (−0.36 to 0.28)	19	0.23 (−0.29 to 0.76)	0.13 (−0.34 to 0.59)	24	−0.17 (−0.46 to 0.12)	−0.14 (−0.58 to 0.31)
Exposed, late pregnancy	50	0.22 (−0.08 to 0.52)	0.08 (−0.19 to 0.35)	17	−0.07 (−0.64 to 0.51)	0.01 (−0.48 to 0.50)	26	−0.00 (−0.65 to 0.65)	0.02 (−0.61 to 0.66)

Abbreviations: ADHD, attention-deficit/hyperactivity disorder; ASQ, Ages and Stages Questionnaires; CPRS-R, Conners’ Parent Rating Scale–Revised.

^a^ The number of exposed pregnancies may differ across the specific outcomes depending on whether these individual measures were reported by mothers.

^b^ The reference group consists of unexposed pregnancies in the corresponding time window.

^c^ Weighted estimates with stabilized inverse probability of treatment weighting (constructed at each time point using baseline covariates, time-varying and time-fixed confounding factors, and benzodiazepine or z-hypnotic treatment history in gestational week 0-16).

**Table 3.** Associations by Class of Medication Exposure on Child Outcomes in 4183 Pregnancies in the Depressive/Anxiety Disorder Stratum^a^

Variable	No.^b^	β (95% CI)	
		Crude Models^c^	Weighted Models^c^^,^^d^
**Benzodiazepine Monotherapy**			
ASQ, gross motor skills			
Exposed, midpregnancy	28	−0.14 (−0.43 to 0.15)	−0.47 (−0.78 to −0.17)
Exposed, late pregnancy	23	0.10 (−0.27 to 0.48)	0.80 (0.12 to 1.48)
ASQ, fine motor skills			
Exposed, midpregnancy	28	0.07 (−0.37 to 0.50)	−0.30 (−0.76 to 0.15)
Exposed, late pregnancy	22	0.19 (−0.26 to 0.63)	0.74 (−0.12 to 1.60)
ASQ, communication skills			
Exposed, midpregnancy	28	0.10 (−0.20 to 0.41)	−0.15 (−0.43 to 0.13)
Exposed, late pregnancy	23	−0.02 (−0.32 to 0.29)	0.06 (−0.16 to 0.27)
CPRS-R, ADHD traits			
Exposed, midpregnancy	27	0.05 (−0.45 to 0.55)	−0.24 (−0.63 to 0.15)
Exposed, late pregnancy	23	0.21 (−0.24 to 0.66)	0.29 (0.02 to 0.57)
**Z-Hypnotic Monotherapy**			
ASQ, gross motor skills			
Exposed, midpregnancy	22	0.21 (−0.35 to 0.77)	0.03 (−0.74 to 0.80)
Exposed, late pregnancy	24	0.54 (−0.05 to 1.13)	0.93 (0.28 to 1.58)
ASQ, fine motor skills			
Exposed, midpregnancy	22	0.54 (−0.02 to 1.10)	0.32 (−0.68 to 1.32)
Exposed, late pregnancy	24	0.78 (0.19 to 1.37)	0.58 (−0.44 to 1.59)
ASQ, communication skills			
Exposed, midpregnancy	21	0.19 (−0.31 to 0.70)	−0.27 (−0.76 to 0.23)
Exposed, late pregnancy	21	0.44 (−0.08 to 0.97)	0.49 (−0.01 to 0.98)
CPRS-R, ADHD traits			
Exposed, midpregnancy	21	0.24 (−0.24 to 0.72)	0.11 (−0.45 to 0.67)
Exposed, late pregnancy	24	0.20 (−0.23 to 0.63)	−0.10 (−0.49 to 0.29)

Abbreviations: ADHD, attention-deficit/hyperactivity disorder; ASQ, Ages and Stages Questionnaires; CPRS-R, Conners’ Parent Rating Scale–Revised.

^a^ Twelve pregnancies were excluded because of co-use of benzodiazepines and z-hypnotics.

^b^ The number of exposed pregnancies may differ across the specific outcomes depending on whether these individual measures were reported by mothers. Overall, there were 68 pregnancies and 50 pregnancies exposed to benzodiazepine monotherapy and z-hypnotic monotherapy at any time in pregnancy, respectively.

^c^ The reference group consists of unexposed pregnancies in the corresponding time window.

^d^ Weighted estimates with stabilized inverse probability of treatment weighting (constructed at each time point using baseline covariates, time-varying and time-fixed confounding factors, and benzodiazepine or z-hypnotic treatment history in gestational week 0-16).

### Associations by Duration of Exposure and Coexposure to Opioids or Antidepressants

Children of mothers with depressive/anxiety disorders who took benzodiazepines/z-hypnotics in multiple 4-week intervals did not show a substantial increased risk for adverse developmental outcomes relative to a sole interval exposed (Table 4). Likewise, coexposure to a benzodiazepine/z-hypnotic-opioid or benzodiazepine/z-hypnotic-antidepressant did not pose any additional risk for the various developmental outcomes than benzodiazepine/z-hypnotic alone.

**Table 4.** Association of Prolonged Benzodiazepine/Z-Hypnotic Use and Coexposure to an Opioid or Antidepressant With Child Developmental Outcomes in the Depressive/Anxiety Disorder Stratum^a^

Variable	No.^b^	β (95% CI)	
		Crude Models	PS-Adjusted Models
**Duration of Benzodiazepine/Z-Hypnotic Exposure**			
ASQ, gross motor skills			
Exposed, 1 interval	82	1 [Reference]	1 [Reference]
Exposed, ≥2 intervals	52	0.05 (−0.31 to 0.40)	−0.05 (−0.44 to 0.34)
ASQ, fine motor skills			
Exposed, 1 interval	81	1 [Reference]	1 [Reference]
Exposed, ≥2 intervals	52	0.33 (−0.03 to 0.69)	0.38 (−0.04 to 0.80)
ASQ, communication skills			
Exposed, 1 interval	79	1 [Reference]	1 [Reference]
Exposed, ≥2 intervals	50	0.17 (−0.21 to 0.54)	0.15 (−0.21 to 0.51)
CPRS-R, ADHD traits			
Exposed, 1 interval	81	1 [Reference]	1 [Reference]
Exposed, ≥2 intervals	51	0.27 (−0.11 to 0.64)	0.35 (−0.11 to 0.81)
**Coexposure to Benzodiazepine/Z-Hypnotic and Opioid**			
ASQ, gross motor skills			
Exposed, benzodiazepine/z-hypnotic	115	1 [Reference]	1 [Reference]
Coexposed, with opioid	19	−0.49 (−0.75 to −0.24)	−0.47 (−0.82 to −0.11)
ASQ, fine motor skills			
Exposed, benzodiazepine/z-hypnotic	114	1 [Reference]	1 [Reference]
Coexposed, with opioid	19	−0.17 (−0.57 to 0.23)	−0.23 (−0.71 to 0.24)
ASQ, communication skills			
Exposed, benzodiazepine/z-hypnotic	110	1 [Reference]	1 [Reference]
Coexposed, with opioid	19	0.29 (−0.14 to 0.72)	0.29 (−0.24 to 0.83)
CPRS-R, ADHD traits			
Exposed, benzodiazepine/z-hypnotic	114	1 [Reference]	1 [Reference]
Coexposed, benzodiazepine/z-hypnotic-opioid	18	−0.18 (−0.58 to 0.23)	−0.30 (−0.83 to 0.22)
**Coexposure to Benzodiazepine/Z-Hypnotic and Antidepressant**			
ASQ, gross motor skills			
Exposed, benzodiazepine/z-hypnotic	82	1 [Reference]	1 [Reference]
Coexposed, with antidepressant	52	0.20 (−0.14 to 0.55)	0.18 (−0.18 to 0.54)
ASQ, fine motor skills			
Exposed, benzodiazepine/z-hypnotic	81	1 [Reference]	1 [Reference]
Coexposed, with antidepressant	52	0.10 (−0.25 to 0.46)	0.06 (−0.34 to 0.46)
ASQ, communication skills			
Exposed, benzodiazepine/z-hypnotic	78	1 [Reference]	1 [Reference]
Coexposed, with antidepressant	51	0.29 (−0.09 to 0.67)	0.27 (−0.16 to 0.69)
CPRS-R, ADHD traits			
Exposed, benzodiazepine/z-hypnotic	80	1 [Reference]	1 [Reference]
Coexposed, with antidepressant	52	0.21 (−0.15 to 0.57)	0.23 (−0.16 to 0.62)

Abbreviations: ADHD, attention-deficit/hyperactivity disorder; ASQ, Ages and Stages Questionnaires; CPRS-R, Conners’ Parent Rating Scale–Revised; PS, propensity score.

^a^ Overall, there were 52 pregnancies exposed in at least 2 intervals vs 82 exposed in 1 interval only. There were 19 pregnancies coexposed to benzodiazepines/z-hypnotics and opioids vs 115 exposed to benzodiazepines/z-hypnotics only. There were 52 pregnancies coexposed to benzodiazepines/z-hypnotics and antidepressants vs 82 exposed to benzodiazepines/z-hypnotics only.

^b^ The number of exposed pregnancies may differ across the specific outcomes depending on whether these individual measures were reported by mothers.

### Associations in Sensitivity Analyses

Our outcome measures were consistently and strongly associated with known predictors or parent report of child medical diagnoses. The negative control was not associated with the various child outcomes except for greater ADHD traits in offspring (eTable 7 and eTable 8 in the Supplement). Results of the sensitivity and probabilistic bias analyses as described in the eAppendix in the Supplement showed that our association measures were generally robust.

## Discussion

This study provides novel evidence on the association between benzodiazepine/z-hypnotic exposure during pregnancy and motor and communication skills and ADHD symptoms in preschoolers. After accounting for time-varying depressive and anxiety symptoms in pregnancy and maternal underlying disorder, we found no substantial increased risk for fine motor deficits or greater ADHD in offspring exposed to benzodiazepine/z-hypnotic medications at different time points in gestation. Although the role of chance, unmeasured factors, and residual confounding by maternal disease severity cannot be ruled out, children of mothers with depressive/anxiety disorders taking a benzodiazepine or z-hypnotic in late gestation had a greater risk for gross motor and communication deficits by age 5 years compared with those unexposed, but not to the extent that the impairment was of clinical relevance.

The association of child ADHD and inherent traits with maternal use of benzodiazepines/z-hypnotics is an underresearched topic.^12^ In 1989, Laegreid et al^39^ described hyperactivity and attention-deficit symptoms in children regularly exposed to benzodiazepine in utero. However, such risk could not be substantiated by recent research, including the present study.^13,14^ Our null association between prenatal benzodiazepine/z-hypnotic use and greater ADHD traits in offspring was consistently observed across the various maternal disorder strata. On the individual drug class level, the negligible association that emerged specifically for benzodiazepine exposure was likely a chance finding or an overestimation of the true drug association due to a failure to correct for exposure misclassification and unmeasured confounding by maternal personality traits and/or familial genetic risk.

Our observed risk for greater gross motor deficits after late pregnancy benzodiazepine/z-hypnotic exposure was evident solely among children of mothers with depressive/anxiety disorders for both drug classes and specific to boys. In absolute terms, we would expect 4 to 6 children to have greater gross motor deficits for every 100 women treated with benzodiazepines or z-hypnotics in late gestation (assuming a 1% prevalence of the outcome among the unexposed).^40,41^ However, the motor proficiency difference herein was below clinically relevant cutoff points^42^ even after accounting for important parental contributors. Although correction for exposure misclassification and unmeasured confounding by maternal personality traits could slightly inflate the difference in motor proficiency between children exposed and unexposed to benzodiazepines/z-hypnotics, this difference would still be below the threshold for a gross motor impairment.^42^ Several factors can explain the lack of finding replication in similarly exposed children born to women with sleeping or pain-related disorders, including residual confounding by maternal psychiatric disease, greater cortisol level and stress in women with depressive/anxiety disorders at the end of gestation, and a higher drug dose regimen in these women.^43,44,45^ Although prior studies on the topic are scarce,^10,11^ interplay between higher drug dose and sex-specific developmental pathways cannot be ruled out.^46^

Disentangling timing from duration or cumulative dose effects is challenging. Unlike prior research,^10,15^ our results do not support the notion that prolonged benzodiazepine/z-hypnotic treatment poses considerable detrimental risks on child motor or communication development relative to shorter-term use.^10^ Our borderline association with fine motor deficits was negligible, with an upper bound below clinically relevant cutoff points for impairment. Although chance findings are possible, our timing and duration results, together with biological research,^47^ can provide some hints about possible mechanisms of developmental alteration by in utero exposure to benzodiazepines/z-hypnotics, as well as its potential interplay with negative perinatal outcomes, such as newborn floppiness, on child motor skills at later age.^46,48^

Albeit with some amount of uncertainty, we observed no strong associations for benzodiazepine/z-hypnotic coexposure to opioid or antidepressant relative to sole benzodiazepine/z-hypnotic use in gestation. Recent research has shown that the risk posed by prenatal antidepressant use on child motor development and ADHD is small in magnitude^49,50^ and most likely attributable to confounding by indication and other unmeasured factors. Our inverse association between benzodiazepine/z-hypnotic–opioid coexposure and child gross motor deficits was an unexpected finding, possibly due to chance and small sample size.

### Limitations

Several limitations of the study need mentioning. Maternal disorders were self-reported, and anxiety was listed only in the prenatal questionnaire at week 17. Depressive and anxiety symptoms were not measured at baseline and were recorded only at 2 time points in pregnancy; however, information about lifetime history of major depression was used in the generation of the stabilized weights. Nondifferential exposure misclassification may be an additional concern that could have led to an underestimation of the true drug associations. Information on dose is not available in the MoBa, which challenges our ability to tease apart timing from duration/cumulative dose effects. Our outcome measures were parent reported; however, their internal consistency was generally satisfactory, and they were strongly associated with known predictors and medical diagnosis of child impairment. Although the risk of outcome misclassification cannot be ruled out, this was probably nondifferential, and the depression distortion bias had a negligible influence on our association estimates. The MoBa has a low response rate (41%), with possible self-selection of the healthiest women.^51^ Although the association measures have been shown to be valid in the MoBa in relation to immediate birth outcomes,^51^ the influence of selection bias on longer-term child outcomes, and thus on our results, cannot be excluded. Our small sample size precluded the duration and coexposure analyses in the sleeping and pain-related disorder strata and limited our detectable effect sizes. Findings of this study may not be generalizable to populations of pregnant women outside Norway.

## Conclusions

We found no substantial increased risk for greater fine motor deficits or ADHD traits in offspring exposed to benzodiazepine/z-hypnotic medications at different time points in gestation or for longer duration. Children born to women with depressive/anxiety disorders who took benzodiazepines and/or z-hypnotics late in pregnancy had greater gross motor and communication deficits compared with the unexposed but not to the extent that the impairment was of clinical relevance. These associations may be attributable to residual confounding by maternal psychiatric disease and/or to a higher-dose drug association in these women, which calls for future dose-effect studies. Prenatal coexposure to a benzodiazepine/z-hypnoyic-opioid or benzodiazepine/z-hypnoyic-antidepressant did not pose any additional detrimental risk on child developmental outcomes at preschool age relative to sole benzodiazepine/z-hypnotic use.

## Notes

Supplement. **eAppendix.** Supplemental Methods **eReferences.** **eFigure 1.** Granularity and Definition of the Exposure Windows, Based on the Timing in Pregnancy When Depressive and Anxiety Symptoms Were Measured **eFigure 2.** Items Composing the Domains of the ASQ and the CRRS-R Instruments in MoBa **eFigure 3.** Distribution of Missing Data on Sufficient Confounders by BZD/Z-Hypnotic Exposure Status in Pregnancy, in Women With Depressive/Anxiety Disorders **eFigure 4.** Distribution of Missing Data on Sufficient Confounders by BZD/Z-Hypnotic Exposure Status in Pregnancy, in Women With Sleeping Problems **eFigure 5.** Distribution of Missing Data on Sufficient Confounders by BZD/Z-Hypnotic Exposure Status in Pregnancy, in Women With Pain-Related Disorders **eTable 1.** Specification of Various Treatment Models in the Marginal Structural Model Analysis **eTable 2.** Timing of Exposure to BZD and Z-Hypnotics, by Maternal Primary Underlying Disorder, With Corresponding Detectable Effect Sizes (d) **eTable 3.** Length of Exposure to BZD and Z-Hypnotics, and Co-exposure With Opioids or Antidepressants, by Maternal Primary Underlying Disorder, With Corresponding Detectable Effect Size (d) **eTable 4.** Cohort Characteristics by Exposure to BZD/Z-Hypnotics During Pregnancy and Maternal Underlying Disorder **eTable 5.** Characteristics of the Generated Stabilized Weights in the Three Maternal Disorder Strata **eTable 6.** Timing Effects of Gestational Exposure to BZDs/Z-Hypnotics on Child Outcomes by Maternal Underlying Disorder, Accounting for Censoring (Pre- and/or Postnatal Loss to Follow-up in MoBa) **eTable 7.** Association of Parent-Reported Dimensional Outcome Measures With Known Predictors or Medical Diagnosis of Child Developmental Delay **eTable 8.** Association of the Negative Control With Child Developmental Outcomes, by Maternal Underlying Disorder
