# Typographical Error in Byline

**DOI:** 10.1001/jamanetworkopen.2019.4291

**Published:** 2019-05-03

In the Original Investigation titled “Association of Maternal Use of Benzodiazepines and Z-Hypnotics During Pregnancy With Motor and Communication Skills and Attention-Deficit/Hyperactivity Disorder Symptoms in Preschoolers,”^1^ a typographical error in Christina D. Chambers’ first name was present on publication but has since been fixed. This article has been corrected.^1^
