# Supplementary material for: Association of Maternal Use of Benzodiazepines and Z-Hypnotics During Pregnancy With Motor and Communication Skills and Attention-Deficit/Hyperactivity Disorder Symptoms in Preschoolers
Source: JAMA Netw Open. 2019 Apr 5;2(4):e191435. doi: 10.1001/jamanetworkopen.2019.1435 (PMC6450329; doi:10.1001/jamanetworkopen.2019.1435)
Supplement: Supplement. — eAppendix. Supplemental Methods eReferences. eFigure 1. Granularity and Definition of the Exposure Windows, Based on the Timing in Pregnancy When Depressive and Anxiety Symptoms Were Measured eFigure 2. Items Composing the Domains of the ASQ and the CRRS-R Instruments in MoBa eFigure 3. Distribution of Missing Data on Sufficient Confounders by BZD/Z-Hypnotic Exposure Status in Pregnancy, in Women With Depressive/Anxiety Disorders eFigure 4. Distribution of Missing Data on Sufficient Confounders by BZD/Z-Hypnotic Exposure Status in Pregnancy, in Women With Sleeping Problems eFigure 5. Distribution of Missing Data on Sufficient Confounders by BZD/Z-Hypnotic Exposure Status in Pregnancy, in Women With Pain-Related Disorders eTable 1. Specification of Various Treatment Models in the Marginal Structural Model Analysis eTable 2. Timing of Exposure to BZD and Z-Hypnotics, by Maternal Primary Underlying Disorder, With Corresponding Detectable Effect Sizes (d) eTable 3. Length of Exposure to BZD and Z-Hypnotics, and Co-exposure With Opioids or Antidepressants, by Maternal Primary Underlying Disorder, With Corresponding Detectable Effect Size (d) eTable 4. Cohort Characteristics by Exposure to BZD/Z-Hypnotics During Pregnancy and Maternal Underlying Disorder eTable 5. Characteristics of the Generated Stabilized Weights in the Three Maternal Disorder Strata eTable 6. Timing Effects of Gestational Exposure to BZDs/Z-Hypnotics on Child Outcomes by Maternal Underlying Disorder, Accounting for Censoring (Pre- and/or Postnatal Loss to Follow-up in MoBa) eTable 7. Association of Parent-Reported Dimensional Outcome Measures With Known Predictors or Medical Diagnosis of Child Developmental Delay eTable 8. Association of the Negative Control With Child Developmental Outcomes, by Maternal Underlying Disorder [file jamanetwopen-2-e191435-s001.pdf]

Supplementary Online Content

Lupattelli A, Chambers CD, Bandoli G, Handal M, Skurtveit S, Nordeng H. Association of maternal use of benzodiazepines and z-hypnotics during pregnancy with motor and communication skills and attention-deficit/hyperactivity disorder symptoms in preschoolers. *JAMA Netw Open*. 2019;2(4):e191435. doi:10.1001/jamanetworkopen.2019.1435

eAppendix. Supplemental Methods

eReferences.

eFigure 1. Granularity and Definition of the Exposure Windows, Based on the Timing in Pregnancy When Depressive and Anxiety Symptoms Were Measured

eFigure 2. Items Composing the Domains of the ASQ and the CRRS-R Instruments in MoBa

eFigure 3. Distribution of Missing Data on Sufficient Confounders by BZD/Z-Hypnotic Exposure Status in Pregnancy, in Women With Depressive/Anxiety Disorders

eFigure 4. Distribution of Missing Data on Sufficient Confounders by BZD/Z-Hypnotic Exposure Status in Pregnancy, in Women With Sleeping Problems

eFigure 5. Distribution of Missing Data on Sufficient Confounders by BZD/Z-Hypnotic Exposure Status in Pregnancy, in Women With Pain-Related Disorders

eTable 1. Specification of Various Treatment Models in the Marginal Structural Model Analysis

eTable 2. Timing of Exposure to BZD and Z-Hypnotics, by Maternal Primary Underlying Disorder, With Corresponding Detectable Effect Sizes (d)

eTable 3. Length of Exposure to BZD and Z-Hypnotics, and Co-exposure With Opioids or Antidepressants, by Maternal Primary Underlying Disorder, With Corresponding Detectable Effect Size (d)

eTable 4. Cohort Characteristics by Exposure to BZD/Z-Hypnotics During Pregnancy and Maternal Underlying Disorder

eTable 5. Characteristics of the Generated Stabilized Weights in the Three Maternal Disorder Strata

eTable 6. Timing Effects of Gestational Exposure to BZDs/Z-Hypnotics on Child Outcomes by Maternal Underlying Disorder, Accounting for Censoring (Pre- and/or Postnatal Loss to Follow-up in MoBa)

eTable 7. Association of Parent-Reported Dimensional Outcome Measures With Known Predictors or Medical Diagnosis of Child Developmental Delay

eTable 8. Association of the Negative Control With Child Developmental Outcomes, by Maternal Underlying Disorder

© 2019 Lupattelli A et al. *JAMA Network Open*.

This supplementary material has been provided by the authors to give readers additional information about their work.

eAppendix, Supplemental Methods

Additional details on "Methods"

Severity of depression and anxiety symptoms

The SCL-25 is a psychometric scale designed to screen for symptoms of depression in population surveys, and it is a reliable instrument as defined by the ICD-10.[1,2] Both SCL-5 and SCL-8 are highly correlated to the SCL-25.[3,4] The SCL-5 and SCL-8 comprise items capturing symptoms of depression and anxiety, such as "Nervousness or shakiness inside", "Feeling fearful", "Suddenly scared for no reason", "Feeling hopeless about the future", and "Feeling everything is an effort". Women could indicate whether they had been bothered by any of the listed symptoms during the last two weeks in a four category response, from "not bothered" to "very bothered", which are rated 1 to 4, respectively. The sum score was calculated separately and divided by the number of items in each instrument.

Main covariates

Co-medication in pregnancy with an opioid was defined as co-use of a drug belonging to the ATC groups N02A. Co-medication with milder analgesics, i.e. non-steroidal anti-inflammatory drugs (NSAIDs) and acetaminophen was defined as co-exposure with a drug belonging to the ATC group M01A or N02BE01, respectively. Likewise, we measured concomitant use of antidepressant medications (ATC group N06A), antipsychotics (ATC group N05A), antiepileptics (ATC group N03A, excluding clonazepam), and sedating antihistamines (ATC R06AD01 and R06AD02). Co-medication was categorized as occurring in early, mid or late pregnancy, or at any time during gestation.

MoBa Q3 measured the number and painfulness of maternal adverse life events, from six months prior to pregnancy to the third trimester. Mothers were asked to indicate whether they had experienced various types of problems (e.g., at work or study place, financial, end of the relationship with the partner), a serious injury or accident, or loss of someone close, and how painful/difficult these events had been (not too bad; painful/difficult; very painful/difficult).

© 2019 Lupattelli A et al. *JAMA Network Open*.

Women were grouped as having experienced “none or at least one not painful adverse life event”, “at least one painful event”, and “at least one very painful event”.

We generated a comorbidity index score on the basis of the study by Bateman et al.[5] The score considered maternal chronic (i.e., congenital heart disease, cardiovascular disorder, lupus, renal disease, chronic hypertension, asthma, diabetes) and pregnancy-specific disorders (i.e. severe preeclampsia, mild preeclampsia, gestational hypertension, and placenta previa), and other correlates (i.e., illicit substance use, daily alcohol use in pregnancy, age, and previous cesarean sections).

Postnatal factors

Length of breastfeeding (in months) was reported by women in MoBa Q4, which measured breastfeeding patterns from birth to when the child was six months old.

When the child was about 5-year old (MoBa Q7), mothers were asked to indicate (as yes/no) whether their children had currently any of the following illnesses confirmed by a doctor or psychologist: i) impaired language development; ii) delayed motor development or clumsy; iii) hearing impairment.

Maternal postnatal symptoms of depression and anxiety were measured in Q4-Q7 via the SCL-8, described earlier.[1-4] We used an average of the SCL scores from early postpartum to when the child was 5-years old.

Adverse life events in the postnatal period were also measured in MoBa Q4-Q7. Mothers were asked to indicate whether they had experienced various types of problems as in MoBa Q3, with the addition of worries that something is wrong with the child. We summed the number of maternal adverse life events in the 0-3 and 4-5 years postpartum.

Maternal and paternal factors

Mothers (in MoBa Q6 at child’s age 3 years) and fathers (MoBa father questionnaire) were asked to complete the short form of the “Adult ADHD Self-Report Scale” (ASRS), a self-report screening scale of adult ADHD.[6] This scale includes six questions concerning the frequency of recent DSM-IV Criterion A - symptoms of adult ADHD. Four questions captured symptoms of

inattention and two questions entailed symptoms of hyperactivity - impulsivity. The five response options range from “never=1” to “very often=5”. We dichotomized the unweighted response options (≤ 2=0, > 3=1) and summed them across the six questions, as indicated in Kessler et al.[6] We then categorized the final ASRS score into three clinical categories (no ADHD symptoms=score 0-1, mild symptoms=score 2-3, moderate to severe symptoms=score 4-6).

Parenting practices were measured via six of the 42-item Alabama Parenting Questionnaire (APQ)[7] in MoBa Q7, i.e. when the child was 5-year old. These selected APQ items measured the “Positive Involvement with children” dimension, which is relevant to the etiology and treatment of child conduct problems.[8] Mothers were asked to indicate on a scale from never=1 to always=5, how often certain situations happened at home (e.g., “You praise your child if he/she behaves well”, “You ask your child about his/her day in childcare”). The item scores were summed, and standardized. Higher z-scores indicate more positive involvement with children.

Fathers were presented with a list of specific disorders and could indicate whether they currently had, or have had in the past, any psychiatric illness, i.e. manic depressive or other long-term mental illnesses, and sleeping problems.

Data analysis

Missing values on the sufficient set of covariates ranged from 0.4-0.7% for maternal and paternal education, to 1.5-1.9% for BMI and alcohol habits, and to 2.1-2.7% for LTH of MD, smoking status and yearly income. For the prenatal SCL-5/8 missing values were 2.8% and 4.6%, and for perinatal history of adverse events they were 4.9%. Overall, 16.5% of the pregnancies had incomplete data in at least one of the confounders. There was no specific pattern of missingness, and we explored the distribution of key variables in relation to missingness, according to BZD/z-hypnotic exposure status (Supplementary Figures 3-5). Under the assumption that data were missing at random, we imputed missing values via multiple imputation with chained equation (ten replications) in each maternal disorder stratum. The imputation procedure included exposure and outcome variables, and auxiliary variables (e.g., maternal age and illnesses, parity, co-medication, risk factors for the outcomes). Imputed data were used in all analyses.[9-11]

In the duration and co-exposure analysis, the propensity score was generated using a modified set of sufficient confounders relative to that used for the main timing analysis: the average standardized score for depressive/anxiety symptoms throughout pregnancy; co-medication with antidepressants, opioids, acetaminophen, or sedating antihistamines, at any time during pregnancy. When exploring co-exposure effects, the co-medication under study was omitted as covariate in the propensity score estimation.

Sensitivity and sub-analyses

We examined the robustness of the main results in the depressive/anxiety disorder stratum in a set of sensitivity analyses, specifically by i) restriction to term pregnancies or offspring with no congenital anomaly; ii) specification of alternative models including additional postnatal, paternal and maternal correlates (Supplementary Table 1). We also tested an interaction term between exposure and offspring sex in the final weighed models. When examining child communication, we excluded children with a medically confirmed hearing impairment, as measured by maternal report.

A complete-case approach was undertaken for the timing, duration, and co-exposure analyses, across the three maternal disorder strata. In the latter analysis, only pregnancy-child dyads with complete information on the sufficient set of confounders were included.

We conducted probabilistic bias analyses to correct for non-differential exposure misclassification, unmeasured confounding, and random error simultaneously (20000 simulations)[12,13] using bias parameters stemming from existing studies,[14,15] across the three maternal disorder strata. To address non-differential misclassification of exposure, we specified a trapezoidal distributions for sensitivity (0.35, 0.50, 0.75, 1.0) and specificity (0.97, 0.98, 0.99, 1.0).[14] To address unmeasured confounding by maternal traits of neuroticism or psychoticism, we assumed the prevalence of these traits to be four time more prevalent in BZD/z-hypnotic exposed (0.20) than unexposed (0.05). We allowed the risk posed by maternal personality traits on child motor development and ADHD to vary between 1.5 and 3.0.[15] For this analysis, we dichotomized our outcome measures (cutoff > 1.5 sd). A combined correction for exposure misclassification and unmeasured confounding was also carried out.[13]

© 2019 Lupattelli A et al. *JAMA Network Open*.

Additional details on "Results"

Sensitivity analysis results

In the depressive/anxiety disorder stratum, restriction to term pregnancies slightly inflated the point estimate for BZD/z-hypnotic late pregnancy exposure on greater gross motor deficits (β_w: 0.78; 95% CI: 0.32 to 1.25), whereas restriction to children with no congenital anomaly did not materially change the main results. When excluding children with impaired hearing at 5 years of age, the association with communication remained, although the CI became wider (β_w =0.40, 95% CI: -0.04, 0.84).

The alternative model specifications accounting for maternal life-time adverse events, child-related postnatal factors, or maternal mental health from delivery to five years postpartum, did not materially change the main results. The association measure between late BZD/z-hypnotic exposure and greater gross motor deficits was reduced (β_w 0.52-0.58) when respectively accounting for paternal disease or maternal depressive and anxiety symptoms at five years postpartum.

There was an interaction between BZD/z-hypnotics in late pregnancy and child's gender in relation to gross motor deficits (β for interaction=-0.84, 95% CI: -1.40 to -0.27; p=0.004). Only boys exposed in late pregnancy to BZD/z-hypnotics had greater gross (β_w: 0.91; 95% CI: 0.47 to 1.35) motor deficits than unexposed. Such association was not evident among girls (β_w: 0.22; 95% CI: -0.19 to 0.63). There was no suggestion of sex-specific differences on the other developmental outcomes, or in the other maternal disorder strata.

The alternative model specification including positive parenting practice by child age five years, produced a stronger relationship between BZD/z-hypnotic late pregnancy exposure and fine motor deficits (β_w: 0.86; 95% CI: 0.15 to 1.57), relative to unexposed. Here, the association measures relating to gross motor and communication skill deficits did not materially change. Upon accounting for maternal and paternal ADHD traits, BZD/z-hypnotic exposure in late pregnancy exerted a protective effect on child greater ADHD traits (β_w: -0.44; 95% CI: -0.89 to 0.01), relative to unexposed. These two latter model specifications were conducted on reduced sample sizes (65% and 35% of the analytical sample, respectively) since parenting practice and parental ADHD traits were only measured in some of the versions of the MoBa questionnaire.

© 2019 Lupattelli A et al. *JAMA Network Open*.

In the complete-case analyses, BZD/z-hypnotic exposure during late gestation seemed to be associated with all child outcomes, including greater ADHD traits ($β_w$=0.34, 95% CI: -0.03, 0.70) and fine motor deficits ($β_w$=0.56, 95% CI: 0.08, 1.05) in the depressive/anxiety disorder stratum. The protective effect of BZD/z-hypnotic in midpregnancy on child communication skills in the sleeping problems stratum was not evident in the complete-case analysis. The protective effect of BZD/z-hypnotic on child fine motor in the pain-related disorder stratum was consistent in the two missing data approaches.

When re-estimating the association measures with a 99% CI, the results of the timing analysis did not materially deviate from the main findings with a 95% CI across the three maternal disorder strata. Children born to women with depressive/anxiety disorders, who took benzodiazepines/z-hypnotics in late pregnancy, had greater motor skill ($β_w$=0.63, 99% CI: 0.06, 1.21) and communication ($β_w$=0.32, 99% CI: -0.06, 0.71) deficits than unexposed in the time window. When replicating the timing analysis by individual drug class, there was no association between benzodiazepine exposure in late pregnancy and greater ADHD symptoms in children ($β_w$=-0.09, 99% CI: -0.59, 0.40).

In the duration analysis the lower bound of the 99% CI of the association between prenatal exposure to benzodiazepines/z-hypnotics in multiple 4-week intervals and greater fine motor skills, moved substantially away from the null ($β_w$=0.37, 99% CI: -0.19, 0.92), relative to the main analysis. The protective effect of prenatal co-exposure to benzodiazepines/z-hypnotics and opioids on child gross motor skills quantified in the main analysis, was no longer evident ($β_w$=-0.57, 99% CI: -1.50, 0.37).

The association measures between BZD/z-hypnotic exposure during pregnancy and child developmental outcomes were shifted further away from the null after correcting for non-differential exposure misclassification and random error. Across the maternal disorder strata, failure to account for exposure misclassification would have underestimated the BZD/z-hypnotic effects of about 12-27% (depression/anxiety disorders), 12-90% (sleeping problems) and 34-69% (pain-related disorders). The bias due to exposure misclassification was lowest in relation to child ADHD traits (12%). When correcting for unmeasured confounding by maternal personality traits and random error, the association measures decreased in magnitude (18% bias) across all strata and for all child outcomes. The combined correction for exposure misclassification,

© 2019 Lupattelli A et al. *JAMA Network Open*.

unmeasured confounding, and random error produced slightly higher association measures in relation to child gross (13% bias) and fine (2% bias) motor skills, and lower association measures (18% bias) with child ADHD traits in the depression/anxiety disorder stratum. Such bias was larger in the remaining strata.

© 2019 Lupattelli A et al. *JAMA Network Open*.

REFERENCES

1. Strand BH, Dalgard OS, Tambs K, Rognerud M. Measuring the mental health status of the Norwegian population: a comparison of the instruments SCL-25, SCL-10, SCL-5 and MHI-5 (SF-36). *Nord J Psychiatry.* 2003;57(2):113-118.
2. Sandanger I, Moum T, Ingebrigtsen G, Dalgard OS, Sorensen T, Bruusgaard D. Concordance between symptom screening and diagnostic procedure: the Hopkins Symptom Checklist-25 and the Composite International Diagnostic Interview I. *Soc Psychiatry Psychiatr Epidemiol.* 1998;33(7):345-354.
3. Fink P, Ornbol E, Huyse FJ, et al. A brief diagnostic screening instrument for mental disturbances in general medical wards. *J Psychosom Res.* 2004;57(1):17-24.
4. Tambs K, Moum T. How well can a few questionnaire items indicate anxiety and depression? *Acta Psychiatr Scand.* 1993;87(5):364-367.
5. Bateman BT, Mhyre JM, Hernandez-Diaz S, et al. Development of a comorbidity index for use in obstetric patients. *Obstet Gynecol.* 2013;122(5):957-965.
6. Kessler RC, Adler L, Ames M, et al. The World Health Organization adult ADHD self-report scale (ASRS): a short screening scale for use in the general population. *Psychol Med.* 2005;35(2):245-256.
7. Javier JR, Coffey DM, Schrager SM, Palinkas LA, Miranda J. Parenting Intervention for Prevention of Behavioral Problems in Elementary School-Age Filipino-American Children: A Pilot Study in Churches. *J Dev Behav Pediatr.* 2016;37(9):737-745.
8. Frick PJ, Christian RE, Wootton JM. Age Trends in the Association between Parenting Practices and Conduct Problems. *Behav Modif.* 1999;23(1):106-128.
9. Rubin DB. *Multiple imputation for nonresponse in surveys.* New York: Wiley; 1987.
10. Sterne JA, White IR, Carlin JB, et al. Multiple imputation for missing data in epidemiological and clinical research: potential and pitfalls. *BMJ.* 2009;338:b2393.
11. Moodie EE, Delaney JA, Lefebvre G, Platt RW. Missing confounding data in marginal structural models: a comparison of inverse probability weighting and multiple imputation. *The international journal of biostatistics.* 2008;4(1):Article 13.
12. Lash TL, Fox MP, Fink AK. *Applying Quantitative Bias Analysis to Epidemiologic Data.* Springer New York; 2009.
13. Orsini N, Bellocco R, Bottai M, Wolk A, Greenland S. A tool for deterministic and probabilistic sensitivity analysis of epidemiologic studies. *Stata Journal.* 2008;8(1):29-48.
14. Skurtveit S, Selmer R, Odsbu I, Handal M. Self-reported data on medicine use in the Norwegian Mother and Child cohort study compared to data from the Norwegian Prescription Database. *Norsk Epidemiologi.* 2014;24(1-2):209-216.
15. Koutra K, Roumeliotaki T, Kyriklaki A, et al. Maternal depression and personality traits in association with child neuropsychological and behavioral development in preschool years: Mother-child cohort (Rhea Study) in Crete, Greece. *J Affect Disord.* 2017;217:89-98.

© 2019 Lupattelli A et al. *JAMA Network Open*.

eFigure 1: Granularity and definition of the exposure windows, based on the timing in pregnancy when depressive and anxiety symptoms were measured*‡

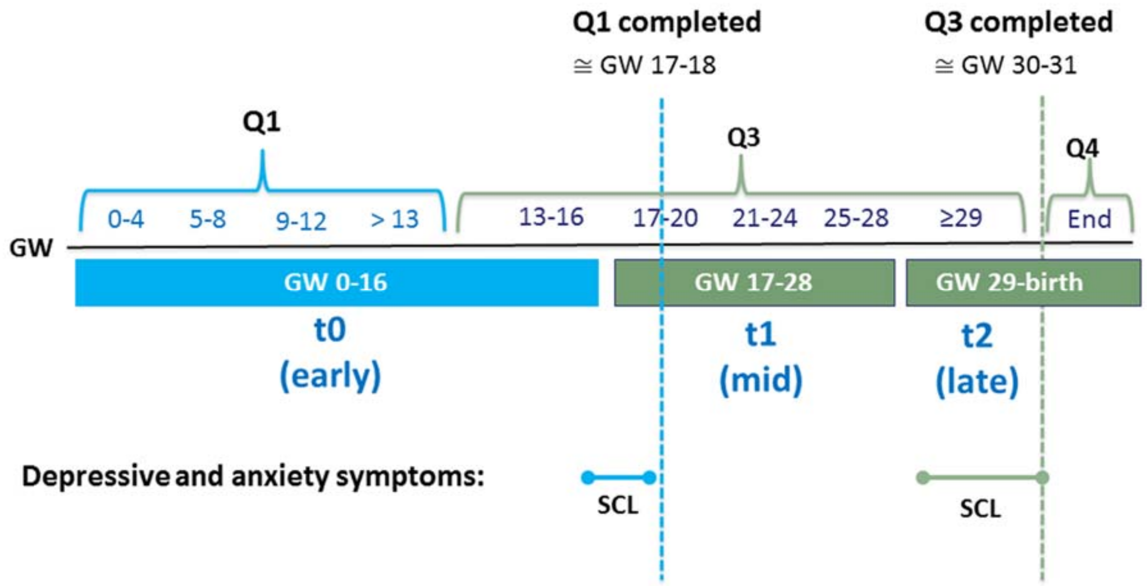

Abbreviations: GW=gestational week. Q1-Q4: MoBa questionnaire 1 (at week 17-18), 3 (at week 30-31) and 4 (at 6 months after childbirth); SCL=Short form of the Hopkins Symptoms Checklist.

*The MoBa questionnaire 2 (at week 22) is not included because it exclusively measured maternal dietary habits.

‡The dotted lines represent the time in pregnancy when maternal symptoms of depression and anxiety were measured upon completion of Q1 and Q3. Q1, which is completed at about week 17-18 in gestation, measures maternal medication use from week 0 to > 13 (blue bracket); Q3, which is completed at about week 30-31 in gestation, measures maternal medication use from week 13 to ≥ 29 (green bracket); Q4 is completed 6 months after birth, and retrospectively measures maternal medication use in the end of pregnancy (green bracket).

eFigure 2 (a, b, c): Items composing the domains of the ASQ and the CRRS-R instruments in MoBa

a: Items in MoBa composing the ASQ 'Gross motor' and 'Fine motor'

ASQ, Gross Motor

1. Walks, runs, and climbs like other children at the same age
2. Able to stand on one foot for at least 5 seconds without problems keeping balance
3. Hops on one foot, many times, without support
4. Plays "catch" with other children; throwing to him/her and catching the ball at least half the time
5. Swings on s swing, pumping by self
6. Rides a two-wheeled bike, with or without training wheels

Response options:
- Yes
- No

ASQ, Fine Motor

1. Puts together a puzzle with nine or more pieces
2. Draws or copies a square with straight corners
3. Cuts with scissors, following a simple outline or pattern
4. Draws pictures of complete people that have at least head: with eyes, nose, mouth; body: arms and legs, hands and feet (need to do all seven for a yes)
5. Colours within the lines in a colouring book
6. Shows interest in and likes to participate in sports or active games requiring good motor skills?

Response options:
- Yes
- No

Abbreviation: ASQ=Ages and Stages Questionnaire.

b: Items in MoBa composing the ASQ ‘Communication’

ASQ, Communication

1. Without giving your child help by pointing or repeating directions, does your child follow three directions that are unrelated to one another? Give all three directions before your child starts. For example, you may ask your child to “Clap your hands, walk to the door, and sit down” or “Give me the pen, open the book, and stand up.”

2. Does your child use four- and five- word sentences? For example, does your child say, “I want the car”?

3. When talking about something that already happened, does your child use words that end in “ed” such as *walked, jumped* or *played*? Ask your child questions, such as “How did you get to the store?” (“We walked.”) “What did you do at your friend’s house?” (“We played.”)

4. Does your child use comparison words, such as *heavier, stronger* or *shorter*? Ask your child questions, such as “A car is *big*, but a bus is _____” (bigger); “A cat is *heavy*, but a man is ____” (heavier); A TV is *small*, but a book is ____” (smaller).

5. Does your child answer the following questions: 1) “What do you do when you are hungry?” (Acceptable answers include: “Get food”, “Eat”, “Ask for something to eat”, and “Have a snack”.) 2) “What do you do when you are tired?” (Acceptable answers include: “Take a nap”, “Rest”, “Go to sleep”, “Go to bed”, “Lie down”, and “Sit down.”)

6. Does your child repeat the sentences shown below back to you, without any mistakes? You may repeat each sentence one time. Mark “yes” if your child repeats both sentences without mistakes or “sometimes” if your child repeats one sentence without mistakes. “Jane hides her shoes for Maria to find.” “Al read the blue book under his bed.”

Response options:

- Yes
- Sometimes
- Not yet

Abbreviation: ASQ=Ages and Stages Questionnaire.

© 2019 Lupattelli A et al. *JAMA Network Open*.

c: Items in MoBa composing the CPRS-R ‘ADHD symptoms’

CPRS-R, ADHD symptoms

1. Inattentive, easily distracted
2. Short attention span
3. Fidgets with hands or feet, squirms in seat
4. Messy or disorganised at home or in the kindergarten
5. Only attends if it is something he/she is very interested in
6. Distractibility or attention span a problem
7. Avoids, expresses reluctance about, or has difficulties engaging in tasks that require sustained mental effort (such as activities in kindergarten or helping out at home)
8. Gets distracted when given instructions to do something
9. Has trouble concentrating in kindergarten
10. Leaves seat in kindergarten or in other situations in which remaining seated is expected
11. Does not follow through on instructions and fails to finish tasks such as putting away shoes/tidying toys (not due to oppositional behaviour or failure to understand instructions)
12. Easily frustrated in efforts

Response options:

- Not true/never/seldom
- Somewhat true/sometimes
- Quite often
- Very often

Abbreviations: CPRS-R=Conners Parent Rating Scale-Revised; ADHD=Attention-deficit/hyperactivity disorder.

eFigure 3: Distribution of missing data on sufficient confounders by BZD/z-hypnotic exposure status in pregnancy, in women with depressive/anxiety disorders

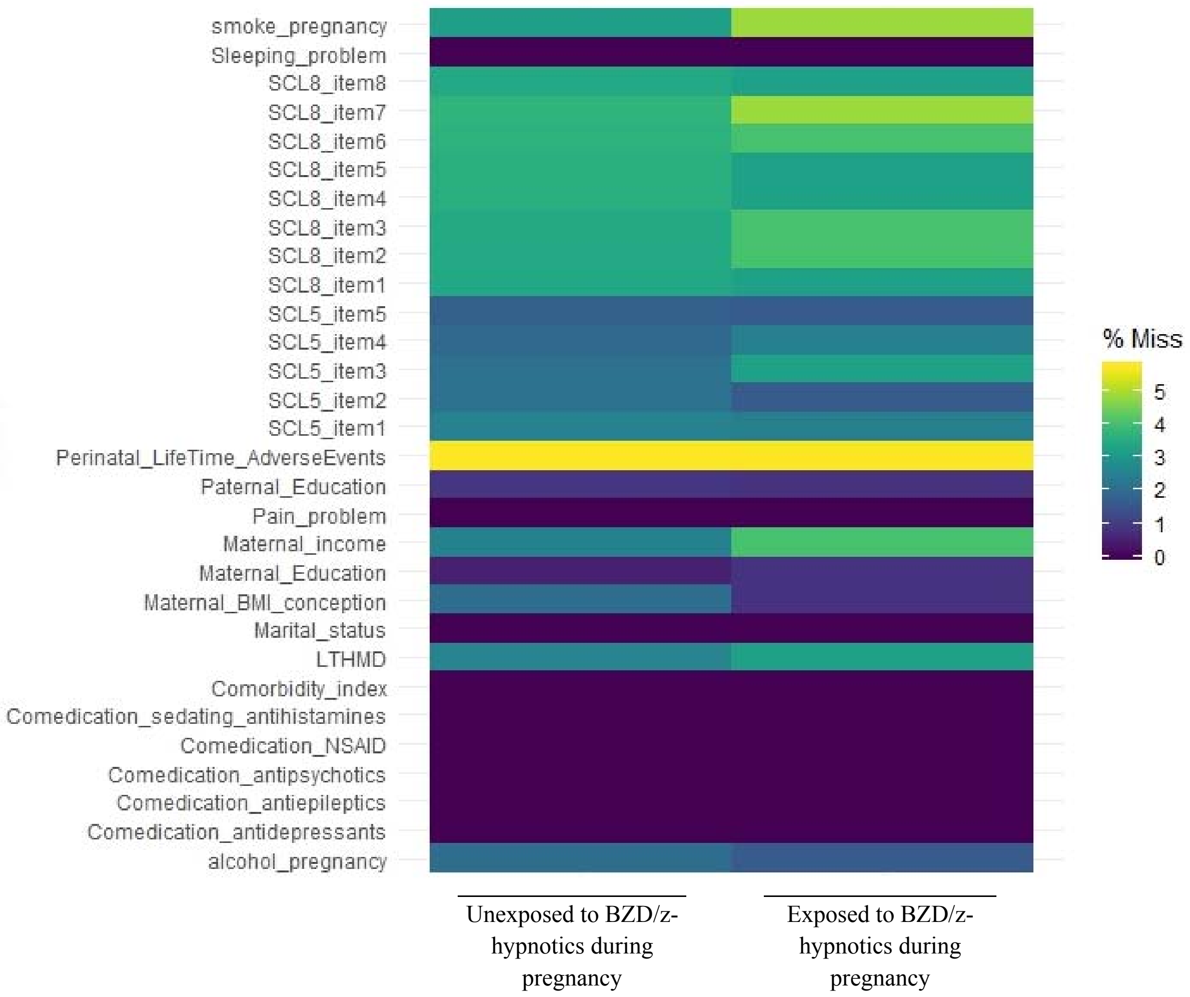

Abbreviations: BDZ=benzodiazepines; BMI=body mass index; SCL-5 and SCL-8=short version (5- and 8-item) of The Hopkins Symptom Checklist; NSAID=non-steroidal anti-inflammatory drugs; LTH of MD=Lite time history of Major Depression.

© 2019 Lupattelli A et al. *JAMA Network Open*.

eFigure 4: Distribution of missing data on sufficient confounders by BZD/z-hypnotic exposure status in pregnancy, in women with sleeping problems

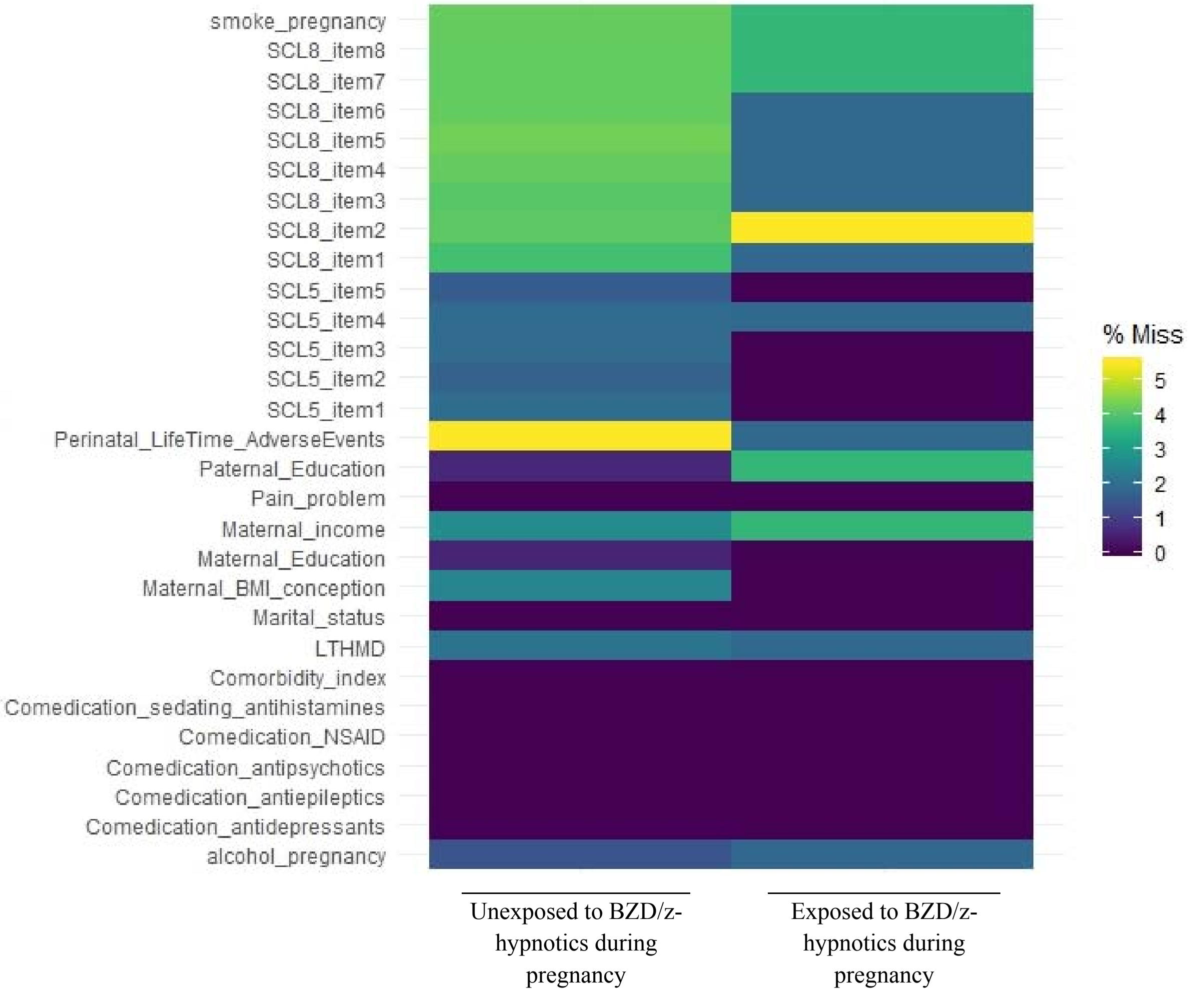

Abbreviations: BDZ=benzodiazepines; BMI=body mass index; SCL-5 and SCL-8=short version (5- and 8-item) of The Hopkins Symptom Checklist; NSAID=non-steroidal anti-inflammatory drugs; LTH of MD=Lite time history of Major Depression.

© 2019 Lupattelli A et al. *JAMA Network Open*.

eFigure 5: Distribution of missing data on sufficient confounders by BZD/z-hypnotic exposure status in pregnancy, in women with pain-related disorders

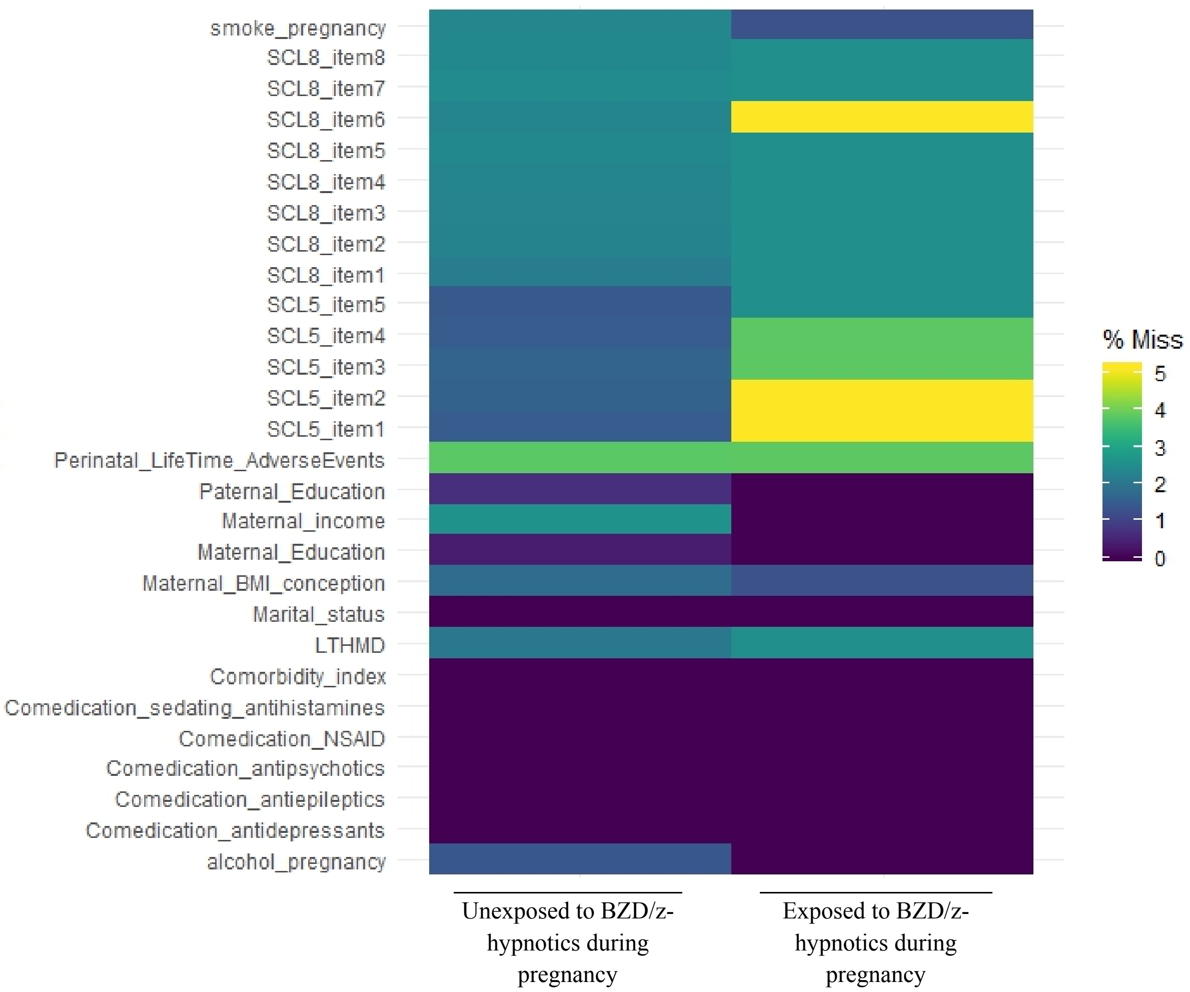

Abbreviations: BDZ=benzodiazepines; BMI=body mass index; SCL-5 and SCL-8=short version (5- and 8-item) of The Hopkins Symptom Checklist; NSAID=non-steroidal anti-inflammatory drugs; LTHMD=Lite time history of Major Depression.

eTable 1: Specification of various treatment models in the marginal structural model analysis

Model	Description
**1**	**IPTW:**<br>**Numerator:**<br>Lagged exposure variable, history of exposure in week 0-16 (T0)<br>**Denominator**:<br>Lagged exposure variable, history of exposure in T0<br>+<br>L: symptoms of depression and lagged variable; co-medication with antidepressants, opioid analgesics, sedative antihistamines, acetaminophen, and lagged variables[‡†]<br>+<br>Z: co-medication with antipsychotic at T0, antiepileptic at T0, NSAID at T0, BMI at conception, maternal education, paternal education, marital status, woman's gross income, perinatal use of folic acid, maternal life-time history of major depression, parity, smoking status at GW 30, alcohol use in pregnancy, comorbidity index, adverse life event from 6 months before to end of pregnancy, pain and/or sleeping problem comorbidity[‡†]
**2**	**IPTW like in model 1, plus risk factors for the outcome/potential intermediates in the denominator:** months of breastfeeding in the 0-6 month period postpartum, child's gender, congenital anomalies, and attendance to daycare/nursery since the child was 13 months old
**3**	**IPTW like in model 1,** but using as history of exposure the time period **6 months prior to pregnancy - end of week 16**
**4**	**IPTW like in model 1, but adding maternal depressive and anxiety symptoms score when the child was 5 year old** to the denominator
**5**	**IPTW like in model 1, but adding maternal depressive and anxiety symptoms score as average from early postpartum to when the child was 5 year old** to the denominator
**6**	**IPTW like in model 1, but adding maternal and paternal ADHD traits** to the denominator
**7**	**IPTW like in model 1, but maternal life-time adverse event** in the 0-3 years postpartum period to the denominator
**8**	**IPTW like in model 1, but maternal life-time adverse event** in the 4-5 years postpartum period to the denominator
**9**	**IPTW like in model 1, but adding paternal age, paternal disease (sleeping problems, psychiatric illness)** to the denominator
**10**	**IPTW like in model 1, but adding positive parenting style** when the child was five year old to the denominator

Abbreviations: IPTW=Inverse Probability of Treatment Weight; GW=gestational week; BMI=body mass index; NSAID=non-steroidal anti-inflammatory drugs; ADHD=attention-deficit/hyperactivity disorder.

© 2019 Lupattelli A et al. *JAMA Network Open*.

Z is a vector of baseline covariates and L of time-varying confounders, including i) depressive and anxiety symptoms as measured at GW 17 and 30; and ii) co-medication during week 0-16 and week 17-28. The specification model 1 is the one chosen and corresponding results are presented as main findings.

[‡]In the pain-related disorder stratum, sleeping and pain comorbidity were not included as variables in the denominator. Co-medication with antipsychotic and antiepileptic at T0, and with antihistamines (time-varying confounder), was not included in the model due to data sparsity. Models 2-10 were not conducted for this stratum.

[†]In the sleeping problems stratum, sleeping comorbidity was not included as variables in the denominator. Alcohol use in pregnancy and co-medication with antidepressants (time-varying confounder) were not included in the model due to data sparsity. Models 2-10 were not conducted for this stratum.

eTable 2: Timing of exposure to BZD and z-hypnotics, by maternal primary underlying disorder, with corresponding detectable effect sizes (d)

Timing of exposure	BZD/z-hypnotic exposed vs. unexposed	BZD exposed vs. unexposed	Z-hypnotic exposed vs. unexposed
	**Depressive and/or anxiety disorders (n=4195)**		
	n exposed (%), d	***n exposed (%), d***	***n exposed (%), d***
Early pregnancy	92 (2.2), d=0.30	53 (1.3), d=0.30	45 (1.1), d=0.30
Mid pregnancy	55 (1.3), d=0.40	32 (0.8), d=0.60	25 (0.6), d=0.60
Late pregnancy	50 (1.2), d=0.40	25 (0.6), d=0.60	27 (0.6), d=0.60
Any time	134 (3.2), d=0.25	80 (1.9), d=0.30	62 (1.5), d=0.60
	**Sleeping problems (n=5260)**		
	n exposed (%), d	***n exposed (%), d***	***n exposed (%), d***
Early pregnancy	45 (0.9), d=0.45	16 (0.3), d=0.70	29 (0.6), d=0.60
Mid pregnancy	19 (0.4), d=0.65	9 (0.2), d=0.95	10 (0.2), d=0.90
Late pregnancy	17 (0.3), d=0.70	6 (0.1), d=1.20	10 (0.2), d=0.90
Any time	60 (1.1), d=0.60	24 (0.5), d=0.60	36 (0.7), d=0.50
	**Pain-related disorders (n=26631)**		
	n exposed (%), d	***n exposed (%), d***	***n exposed (%), d***
Early pregnancy	50 (0.2), d=0.40	28 (0.1), d=0.60	19 (0.1), d=0.65
Mid pregnancy	25 (0.1), d=0.60	12 (0.1), d=0.80	12 (0.1), d=0.80
Late pregnancy	26 (0.1), d=0.60	16 (0.1), d=0.70	10 (0.04), d=0.90
Any time	89 (0.3), d=0.30	52 (0.2), d=0.40	35 (0.1), d=0.50

Abbreviations: BDZ=benzodiazepines; d=detectable effect size.

Assumed 80% power (α = 0.05), and varying allocation ratios. Power calculation was aided by GPower using mean difference test between two independent groups (Faul F, Erdfelder E, Buchner A, Lang AG. Statistical power analyses using G*Power 3.1: tests for correlation and regression analyses. Behav Res Methods 2009;41:1149-60).

eTable 3: Length of exposure to BZD and z-hypnotics, and co-exposure with opioids or antidepressants, by maternal primary underlying disorder, with corresponding detectable effect size (d)

	n (%) exposed vs. n (%) comparator, d
**Depressive and/or anxiety disorders (n=4195)**	
≥2 intervals duration vs 1 interval	52 (1.2) vs 82 (2.0), d=0.52
Opioid co-exposed vs BZD/z-hypnotics only	19 (0.5) vs 115 (2.7), d=0.75
Antidepressant co-exposed vs BZD/z-hypnotics only	52 (1.2) vs 82 (2.0), d=0.52
**Sleeping problems (n=5260)**	
≥2 intervals duration vs 1 interval	25 (0.5) vs 35 (0.7), d=0.80
Opioid co-exposed vs BZD/z-hypnotics only	5 (0.1) vs 55 (1.1), -
Antidepressant co-exposed vs BZD/z-hypnotics only	1 (0.02) vs 59 (1.1), -
**Pain-related disorders (n=26631)**	
≥2 intervals duration vs 1 interval	21 (0.1) vs 68 (0.3), d=0.75
Opioid co-exposed vs BZD/z-hypnotics only	11 (0.04) vs 78 (0.3), d=0.95
Antidepressant co-exposed vs BZD/z-hypnotics only	2 (0.01) vs 87 (0.3), -

Abbreviations: BDZ=benzodiazepines; d=detectable effect size.

Assumed 80% power (α = 0.05), and varying allocation ratios. Power calculation was aided by GPower using mean difference test between two independent groups (Faul F, Erdfelder E, Buchner A, Lang AG. Statistical power analyses using G*Power 3.1: tests for correlation and regression analyses. Behav Res Methods 2009;41:1149-60).

eTable 4: Cohort characteristics by exposure to BZD/z-hypnotics during pregnancy and maternal underlying disorder

Characteristics	Depressive/anxiety disorder, n=4195		Sleeping problems, n=5260		Pain-related disorders, n=26631	
	BZD/z-hypnotic exposure during pregnancy†					
	No N=4061	Yes N=134	No N=5200	Yes N=60	No N=26542	Yes N=89
Maternal sociodemographic, life-style characteristics						
**Age (years); mean ± sd**	30.6±4.8	31.6±4.3	30.9±4.5	32.1±4.4	30.5±4.3	31.6±4.5
**BMI at conception; mean ± sd**	24.0±4.3	23.8±4.2	23.9±4.2	24.3±4.7	24.0±4.1	23.6±3.7
**Primiparous; n (%)**	2101 (51.7)	70 (52.2)	2567 (49.3)	27 (45.0)	12284 (46.3)	46 (51.7)
**Married/Cohabiting; n (%)**	3790 (93.3)	119 (88.8)	5000 (96.2)	56 (93.3)	25781 (97.1)	83 (93.3)
**Educational level;[a] n (%)**						
University/College	2609 (64.3)	94 (70.2)	3707 (71.3)	48 (80.0)	19424 (73.2)	69 (77.5)
Lower than University/College	1450 (35.7)	40 (29.9)	1490 (28.7)	12 (20.0)	7110 (26.8)	20 (22.5)
**Gross yearly income;[b] n (%)**						
Average	2821 (71.2)	102 (79.7)	3726 (73.6)	39 (67.2)	19800 (76.6)	63 (70.8)
Low	707 (17.9)	16 (12.5)	600 (11.9)	6 (10.3)	2704 (10.5)	11 (12.4)
High	433 (10.9)	10 (7.8)	739 (14.6)	13 (22.4)	3343 (12.9)	15 (16.9)
**Smoking status (yes) at week 30; n (%)**	329 (8.1)	21 (15.7)	224 (4.3)	6 (10.0)	1081 (4.1)	7 (7.9)
**Alcohol use in pregnancy; n (%)**						
No/very limited use	3501 (86.2)	107 (80.0)	4643 (89.3)	53 (88.3)	23926 (90.1)	65 (73.0)
Medium use	490 (12.1)	21 (15.7)	506 (9.7)	7 (11.7)	2398 (9.0)	18 (20.2)
Weekly use	62 (1.5)	6 (4.5)	44 (0.9)	-	194 (0.7)	6 (6.7)
**Folate intake[c] (yes); n (%)**	3572 (88.0)	119 (88.8)	4600 (88.5)	52 (86.7)	23327 (87.9)	79 (88.8)
Maternal health characteristics						
**Comorbidity index; mean ± sd**	0.10±1.06	0.37±1.16	0.08±1.04	0.18±1.23	-0.00±1.00	0.43±1.36
**LTH of MD[d] (yes); n (%)**	1070 (26.4)	47 (35.1)	285 (5.5)	3 (5.0)	931 (3.5)	7 (7.9)
**Depressive/anxiety symptoms during pregnancy; z-score ± sd**						
SCL-5 at week 17	0.04±1.01	0.51±1.30	0.11±1.08	0.44±1.32	0.09±1.10	0.34±1.61
SCL-8 at week 30	0.03±1.02	0.50±1.24	0.11±1.09	0.47±1.40	0.09±1.08	0.44±1.37
**Emotional stability trait (range 1-5);[e] mean ± sd**	2.99±0.53	3.04±0.47	2.75±0.54	2.83±0.51	2.67±0.51	2.81±0.52

© 2019 Lupattelli A et al. *JAMA Network Open*.

Characteristics	Depressive/anxiety disorder, n=4195		Sleeping problems, n=5260		Pain-related disorders, n=26631	
	BZD/z-hypnotic exposure during pregnancy[†]					
	No N=4061	Yes N=134	No N=5200	Yes N=60	No N=26542	Yes N=89
**Life-time adverse event at baseline;[e] n (%)**						
None or at least one event but not painful	1750 (43.3)	42 (31.3)	3152 (60.9)	24 (40.0)	17931 (67.7)	50 (56.2)
At least one event, painful	1242 (30.8)	49 (36.6)	1313 (25.4)	22 (36.7)	5966 (22.5)	27 (30.3)
At least one event, very painful	1047 (25.9)	43 (32.1)	712 (13.8)	14 (23.3)	2574 (9.7)	12 (13.5)
**Co-medication in pregnancy (yes); n (%)**						
Antidepressants	338 (8.3)	52 (38.8)	10 (0.2)	1 (1.7)	13 (0.1)	2 (2.3)
Antipsychotics	64 (1.6)	11 (8.2)	52 (1.0)	4 (6.7)	168 (0.6)	2 (2.3)
Opioid analgesics	123 (3.0)	19 (14.2)	134 (2.6)	5 (8.3)	450 (1.7)	11 (12.4)
Antiepileptic drugs	27 (0.7)	2 (1.5)	19 (0.4)	3 (5.0)	87 (0.3)	1 (1.1)
NSAIDs	358 (8.8)	20 (14.9)	387 (7.4)	10 (2.5)	1624 (6.1)	11 (12.4)
Acetaminophen	2174 (53.3)	92 (68.7)	2679 (51.5)	41 (68.3)	12718 (47.9)	64 (71.9)
Sedating antihistamines	39 (1.0)	15 (11.2)	42 (0.8)	8 (13.3)	101 (0.4)	2 (2.3)
**Illicit substance use[g] (yes); n (%)**	63 (1.6)	10 (7.5)	35 (0.7)	-	104 (0.4)	4 (4.5)
Child's and postpartum characteristics						
**Breastfeeding months up to child's age 6 months; mean ± sd**	5.4 ± 2.6	5.4 ± 2.6	5.5 ± 2.5	5.5 ± 2.4	5.7 ± 2.4	5.7 ± 2.4
**Infant gender (male); n (%)**	2043 (50.3)	64 (47.8)	2580 (49.6)	25 (41.7)	13522 (51.0)	46 (51.7)
**Any malformation (yes); n (%)**	206 (5.1)	10 (7.5)	257 (4.9)	1 (1.7)	1282 (4.8)	2 (2.3)
**Premature birth (yes); n (%)**	212 (5.2)	6 (4.6)	263 (5.1)	3 (5.0)	1142 (4.3)	8 (9.0)
**Nursery/daycare attendance; n (%)**						
Never between 1-5 years of age	622 (15.3)	24 (17.9)	806 (15.5)	6 (10.0)	3801 (14.3)	20 (22.5)
Any time between 1-5 years of age	2954 (72.7)	92 (68.7)	3726 (71.7)	47 (78.3)	19030 (71.7)	59 (66.3)
Always between 1-5 years of age	485 (11.9)	18 (13.4)	668 (12.9)	7 (11.7)	3711 (14.0)	10 (11.2)
**Number of postnatal maternal adverse events;[h] mean ± sd**						
Between 0-3 years postpartum	0.89±1.18	1.00±1.20	0.67±1.01	0.75±1.02	0.53±0.88	0.73±1.06
Between 4-5 years postpartum	1.22±1.31	1.58±1.47	0.91±1.11	1.13±1.23	0.72±0.99	0.91±1.23
**Postnatal depressive/anxiety symptoms; z-score ± sd**						
SCL-8 average between 0.5-5 year postpartum	0.06±1.00	0.37±1.07	0.10±1.09	0.49±1.38	0.10±1.08	0.60±1.46
SCL-8 specifically at 5 years postpartum	0.03±1.02	0.16±1.01	0.08±1.09	0.42±1.18	0.07±1.07	0.45±1.50

© 2019 Lupattelli A et al. *JAMA Network Open*.

Characteristics	Depressive/anxiety disorder, n=4195		Sleeping problems, n=5260		Pain-related disorders, n=26631	
	BZD/z-hypnotic exposure during pregnancy†					
	No N=4061	Yes N=134	No N=5200	Yes N=60	No N=26542	Yes N=89
**Maternal ADHD symptom level at 3 year postpartum; n (%)**						
None	2595 (63.9)	83 (61.9)	3724 (71.6)	47 (78.3)	20080 (75.7)	60 (67.4)
Mild	536 (13.2)	21 (15.7)	374 (7.2)	3 (5.0)	1362 (5.1)	8 (9.0)
Moderate to severe	108 (2.7)	6 (4.5)	63 (1.2)	-	179 (0.7)	2 (2.3)
**Parental positive involvement with child; z-score ± sd**	0.00 (0.99)	-0.16 (1.21)	0.01 (1.00)	-0.43 (1.1)	-0.00 (1.00)	0.16 (0.91)
Paternal characteristics						
**Age (years); n (%)**						
< 25	204 (5.0)	4 (3.0)	201 (3.9)	1 (1.7)	850 (3.2)	-
25-39	3344 (82.3)	103 (76.9)	4406 (84.7)	52 (86.7)	23036 (86.8)	72 (80.9)
40-49	456 (11.2)	24 (17.9)	558 (10.7)	5 (8.3)	2408 (9.1)	14 (15.7)
> 49	44 (1.1)	24 (0.8)	25 (0.5)	1 (1.7)	188 (0.7)	1 (1.1)
**Educational level; n (%)**						
University/College	2002 (49.3)	68 (50.8)	2815 (54.1)	29 (48.3)	14455 (54.5)	50 (56.2)
Lower than University/College	2057 (50.7)	66 (49.3)	2382 (45.8)	31 (51.7)	12073 (45.5)	39 (43.8)
**Sleeping problems (yes); n (%)**	311 (7.7)	10 (7.5)	299 (5.6)	6 (10.0)	1129 (4.3)	7 (7.9)
**Mental illness (yes); n (%)**	86 (2.1)	2 (1.5)	59 (1.1)	-	245 (0.9)	2 (2.3)
**LTH of MD[d] (yes); n (%)**	535 (13.2)	14 (10.5)	467 (9.0)	6 (10.0)	2007 (7.6)	10 (11.2)
**Paternal ADHD symptom at time of pregnancy; n (%)**						
None	1444 (79.6)	47 (83.9)	2024 (84.0)	21 (77.8)	10192 (85.7)	33 (76.7)
Mild	318 (17.5)	8 (14.3)	354 (14.7)	5 (18.5)	1529 (12.8)	9 (20.9)
Moderate to severe	52 (2.9)	1 (1.8)	31 (1.3)	1 (3.7)	175 (1.5)	1 (2.4)

Abbreviations: BDZ=benzodiazepines; BMI=body mass index; SCL-5 and SCL-8=short version (5- and 8-item) of The Hopkins Symptom Checklist; NSAID=non-steroidal anti-inflammatory drugs; LTH of MD=Lite time history of Major Depression; ADHD=Attention Deficit Hyperactive Disorder.

†Numbers may not add up to total due to missing values, ranging from 0.4-0.7% (maternal/paternal education), to 1.5-1.9% (BMI , alcohol habits), and 2.1-2.7% (LTH of MD, smoking status and income). For the prenatal SCL-5/8 missing values were 2.8% and 4.6%, and for perinatal history of adverse events they were 4.9%. Information on maternal emotional stability traits, maternal and paternal ADHD, and parenting, was available for 30-65% of the study population because the instruments were only present in later versions of the MoBa questionnaire.

[a]Ongoing or completed educational level. [b]Average: 14800 to 49900 USD; low: ≤ 14800 USD; high: ≥ 50000 USD. [c]Folate before and/or during first trimester. [d]Defined as Kendlers Life time major depression scale score of 3 or more simultaneous depressive symptoms of duration of more than 2 weeks. [e]As measured by the International Personality Item Pool (IPIP) Big-Five Factor Markers. [f]Adverse life events in the perinatal period, i.e. from 7 months before pregnancy to week 30 of pregnancy. [g]Before and/or during pregnancy. [h]Number of adverse life events in the early and late postnatal period, with no severity specification.

© 2019 Lupattelli A et al. *JAMA Network Open*.

eTable 5: Characteristics of the generated stabilized weights in the three maternal disorder strata

	Depressive/anxiety disorder, n=4195	Sleeping problems, n=5260	Pain-related disorders, n=26631
**IPTW**			
Mean, SD	1.00, 0.17	1.00, 0.08	1.00, 0.05
Min-Max	0.09-8.85	0.07-4.41	0.03-6.06
**IPTW*IPCW[a]**			
Mean, SD	0.98, 0.17	0.97, 0.09	0.97, 0.06
Min-Max	0.09-8.64	0.07-4.21	0.03-5.71
**IPTW*IPCW[b]**			
Mean, SD	0.96, 0.18	0.95, 0.09	0.94, 0.06
Min-Max	0.10-9.72	0.07-4.16	0.03-5.46

Abbreviations: IPTW: inverse probability of treatment weight; IPCW: inverse probability of censoring weight; SD=standard deviation.

[a]Stabilized inverse probability of censoring weighting at 5 years postpartum. [b]Stabilized inverse probability of censoring weighting in pregnancy (at gestational week 30), at 6 months postpartum, and at 5 years postpartum.

eTable 6: Timing effects of gestational exposure to BZDs/z-hypnotics on child outcomes by maternal underlying disorder, accounting for censoring (pre- and/or postnatal loss to follow-up in MoBa)

	Maternal primary underlying disorder					
	**Depressive/anxiety disorder n=4195**		**Sleeping problems n=5260**		**Pain-related disorders n=26631**	
**BZD/z-hypnotic**	**Weighted[a,b] β (95% CI)**	**Weighted[a,c] β (95% CI)**	**Weighted[a,b] β (95% CI)**	**Weighted[a,c] β (95% CI)**	**Weighted[a,b] β (95% CI)**	**Weighted[a,c] β (95% CI)**
**ASQ, gross motor skills**						
Exposed, midpregnancy	-0.19 (-0.55, 0.17)	-0.20 (-0.57, 0.16)	-0.30 (-0.51, -0.08)	-0.29 (-0.51, -0.08)	0.17 (-0.70, 1.03)	0.17 (-0.70, 1.04)
Exposed, late pregnancy	0.67 (0.21, 1.13)	0.70 (0.21, 1.19)	-0.20 (-0.55, 0.16)	-0.20 (-0.55, 0.14)	-0.05 (-0.67, 0.57)	-0.05 (-0.66, 0.57)
**ASQ, fine motor skills**						
Exposed, midpregnancy	0.28 (-0.05, 0.61)	0.03 (-0.44, 0.50)	-0.21 (-0.69, 0.27)	-0.21 (-0.69, 0.27)	0.07 (-0.33, 0.46)	0.07 (-0.33, 0.47)
Exposed, late pregnancy	0.53 (0.15, 0.90)	0.52 (-0.11, 1.16)	0.06 (-0.59, 0.71)	0.05 (-0.59, 0.70)	-0.44 (-0.69, -0.20)	-0.44 (-0.69, -0.19)
**ASQ, communication skills**						
Exposed, midpregnancy	-0.11 (-0.40, 0.19)	-0.10 (-0.39, 0.19)	0.19 (-0.29, 0.68)	0.20 (-0.29, 0.68)	-0.17 (-0.58, 0.23)	-0.17 (-0.58, 0.24)
Exposed, late pregnancy	0.34 (0.04, 0.64)	0.32 (0.03, 0.62)	-0.27 (-0.63, 0.08)	-0.28 (-0.63, 0.06)	-0.05 (-0.46, 0.34)	-0.05 (-0.46, 0.36)
**CPRS-R, ADHD traits**						
Exposed, midpregnancy	-0.04 (-0.36, 0.29)	-0.04 (-0.36, 0.28)	0.11 (-0.35, 0.57)	0.10 (-0.36, 0.56)	-0.15 (-0.59, 0.30)	-0.15 (-0.59, 0.30)
Exposed, late pregnancy	0.08 (-0.19, 0.36)	0.09 (-0.18, 0.36)	0.02 (-0.48, 0.52)	0.02 (-0.48, 0.52)	0.04 (-0.60, 0.69)	0.05 (-0.60, 0.70)

Abbreviations: BDZ=benzodiazepines; ASQ=Ages and Stages Questionnaire; CPRS-R= Conners Parent Rating Scale-Revised; ADHD= attention-deficit/hyperactivity disorder. [a]Reference: unexposed pregnancies in the corresponding time window. [b]Weighted estimates with stabilized inverse probability of treatment weighting (constructed at each time point using baseline covariates, time-varying and time-fixed confounding factors, and BZD/z-hypnotic history treatment) and censoring weighting (loss to follow-up at 5 years postpartum). [c]Weighted estimates with stabilized inverse probability of treatment weighting (constructed as described in b) and censoring weighting during gestation (loss to follow-up at gestational week 30) and postnatally (loss to follow-up at six months and 5 years postpartum).

© 2019 Lupattelli A et al. *JAMA Network Open*.

eTable 7: Association of parent-reported dimensional outcome measures with known predictors or medical diagnosis of child developmental delay

	ASQ, gross motor skills<br><br>Crude β (95% CI)	ASQ, fine motor skills<br><br>Crude β (95% CI)	ASQ, communication skills<br><br>Crude β (95% CI)	CPRS-R, ADHD traits<br><br>Crude β (95% CI)
**Depressive/anxiety disorder, n=4195**				
**Diagnosis of child delayed motor/clumsy**				
No	Reference	Reference		
Yes	3.31 (2.85, 3.77)	1.96 (1.53, 2.39)		
**Diagnosis of child impaired language development**				
No			Reference	
Yes			2.29 (1.62, 2.96)	
**Maternal ADHD symptoms**				
None				Reference
Mild				0.41 (0.31, 0.52)
Moderate to severe				0.66 (0.42, 0.89)
**Paternal ADHD symptoms**				
None				Reference
Mild				0.18 (0.05, 0.31)
Moderate to severe				0.30 (-0.01, 0.61)
**Sleeping problems, n=5260**				
**Diagnosis of child delayed motor/clumsy**				
No	Reference	Reference		
Yes	4.33 (3.79, 4.88)	2.45 (2.02, 2.89)		
**Diagnosis of child impaired language development**				
No			Reference	
Yes			2.06 (1.51, 2.61)	
**Maternal ADHD symptoms**				
None				Reference
Mild				0.53 (0.39, 0.66)
Moderate to severe				0.61 (0.23, 0.99)
**Paternal ADHD symptoms**				
None				Reference
Mild				0.12 (0.00, 0.24)
Moderate to severe				0.12 (-0.18, 0.41)
**Pain-related disorders, n=26631**				
**Diagnosis of child delayed motor/clumsy**				
No	Reference	Reference		
Yes	4.48 (4.11, 4.85)	2.46 (2.19, 2.73)		
**Diagnosis of child impaired language development**				
No			Reference	
Yes			2.45 (2.13, 2.76)	

	ASQ, gross motor skills<br><br>Crude β (95% CI)	ASQ, fine motor skills<br><br>Crude β (95% CI)	ASQ, communi-cation skills<br><br>Crude β (95% CI)	CPRS-R, ADHD traits<br><br>Crude β (95% CI)
**Depressive/anxiety disorder, n=4195**				
**Maternal ADHD symptoms**				
None				Reference
Mild				0.51 (0.44, 0.58)
Moderate to severe				0.76 (0.54, 0.98)
**Paternal ADHD symptoms**				
None				Reference
Mild				0.13 (0.07, 0.19)
Moderate to severe				0.25 (0.07, 0.43)

Abbreviations: BDZ=benzodiazepines; ASQ=Ages and Stages Questionnaire; CPRS-R= Conners Parent Rating Scale-Revised; ADHD= attention-deficit/hyperactivity disorder.

eTable 8: Association of the negative control with child developmental outcomes, by maternal underlying disorder

	Maternal primary underlying disorder					
	Depressive/anxiety disorder n=4061		Sleeping problems n=5200		Pain-related disorders n=26542	
	Crude β (95% CI)	PS adjusted[a] β (95% CI)	Crude β (95% CI)	PS adjusted[a] β (95% CI)	Crude β (95% CI)	PS adjusted[a] β (95% CI)
**ASQ, gross motor skills**						
BZD/z-hypnotic exposure only before pregnancy	-0.01 (-0.17, 0.15)	-0.03 (-0.19, 0.13)	0.03 (-0.36, 0.41)	0.00 (-0.37, 0.38)	0.11 (-0.19, 0.41)	0.09 (-0.21, 0.39)
**ASQ, fine motor skills**						
BZD/z-hypnotic exposure only before pregnancy	0.04 (-0.17, 0.24)	0.05 (-0.16, 0.26)	0.30 (-0.14, 0.73)	0.28 (-0.15, 0.71)	0.06 (-0.21, 0.33)	0.04 (-0.22, 0.30)
**ASQ, communication skills**						
BZD/z-hypnotic exposure only before pregnancy	-0.07 (-0.26, 0.11)	-0.04 (-0.23, 0.15)	0.08 (-0.24, 0.40)	0.10 (-0.22, 0.42)	0.10 (-0.16, 0.37)	0.09 (-0.18, 0.36)
**CPRS-R, ADHD traits**						
BZD/z-hypnotic exposure only before pregnancy	0.19 (-0.03, 0.41)	0.15 (-0.08, 0.37)	0.13 (-0.22, 0.48)	0.10 (-0.25, 0.45)	0.31 (0.04, 0.58)	0.27 (0.01, 0.53)

Abbreviations: BDZ=benzodiazepines; ASQ=Ages and Stages Questionnaire; CPRS-R= Conners Parent Rating Scale-Revised; ADHD= attention-deficit/hyperactivity disorder; PS=Propensity score.

The reference group consists of pregnancies within women unexposed to BZD/z-hypnotics both before and during pregnancy.
[a]The PS was estimated using baseline maternal covariates.
